# Terpene Moiety Enhancement by Overexpression of Geranyl(geranyl) Diphosphate Synthase and Geraniol Synthase Elevates Monomeric and Dimeric Monoterpene Indole Alkaloids in Transgenic *Catharanthus roseus*

**DOI:** 10.3389/fpls.2018.00942

**Published:** 2018-07-06

**Authors:** Sarma R. Kumar, H. B. Shilpashree, Dinesh A. Nagegowda

**Affiliations:** Molecular Plant Biology and Biotechnology Lab, Research Centre, CSIR-Central Institute of Medicinal and Aromatic Plants, Bengaluru, India

**Keywords:** *Catharanthus roseus*, geraniol synthase, geranyl(geranyl) diphosphate synthase, overexpression, monoterpene indole alkaloids, transgenic plant

## Abstract

*Catharanthus roseus* is the sole source of two of the most important anticancer monoterpene indole alkaloids (MIAs), vinblastine and vincristine and their precursors, vindoline and catharanthine. The MIAs are produced from the condensation of precursors derived from indole and terpene secoiridoid pathways. It has been previously reported that the terpene moiety limits MIA biosynthesis in *C. roseus*. Here, to overcome this limitation and enhance MIAs levels in *C. roseus*, bifunctional geranyl(geranyl) diphosphate synthase [G(G)PPS] and geraniol synthase (GES) that provide precursors for early steps of terpene moiety (secologanin) formation, were overexpressed transiently by agroinfiltration and stably by *Agrobacterium*-mediated transformation. Both transient and stable overexpression of *G(G)PPS* and co-expression of *G(G)PPS*+*GES* significantly enhanced the accumulation of secologanin, which in turn elevated the levels of monomeric MIAs. In addition, transgenic *C. roseus* plants exhibited increased levels of root alkaloid ajmalicine. The dimeric alkaloid vinblastine was enhanced only in *G(G)PPS* but not in *G(G)PPS*+*GES* transgenic lines that correlated with transcript levels of peroxidase-1 (*PRX1*) involved in coupling of vindoline and catharanthine into 3′,4′-anhydrovinblastine, the immediate precursor of vinblastine. Moreover, first generation (T_1_) lines exhibited comparable transcript and metabolite levels to that of T_0_ lines. In addition, transgenic lines displayed normal growth similar to wild-type plants indicating that the bifunctional G(G)PPS enhanced flux toward both primary and secondary metabolism. These results revealed that improved availability of early precursors for terpene moiety biosynthesis enhanced production of MIAs in *C. roseus* at the whole plant level. This is the first report demonstrating enhanced accumulation of monomeric and dimeric MIAs including root MIA ajmalicine in *C. roseus* through transgenic approaches.

## Introduction

*Catharanthus roseus* (Madagascar Periwinkle) is the best-characterized monoterpene indole alkaloids (MIAs)-producing plant species. To date, *C. roseus* remains the only natural source of two medicinally valuable dimeric MIAs vinblastine and vincristine, and their monomeric precursors vindoline and catharanthine (Miettinen et al., 2014; Kellner et al., 2015). While leaf specific dimeric alkaloids vinblastine and vincristine are used either directly or as derivatives in cancer chemotherapy, roots accumulate monomeric alkaloids ajmalicine and serpentine which are used as anti-hypertensive agents (Zhao et al., 2013). Coupling of the monomeric precursors vindoline and catharanthine to α-3′,4′-anhydrovinblastine and its subsequent conversion results in the formation of dimeric vinblastine and vincristine (Costa et al., 2008). Extremely low *in planta* accumulation of dimeric alkaloids makes them highly expensive. Approximately 500 kg of dry leaves is required to isolate 1 g of vinblastine for pharmaceutical production (Noble, 1990). Moreover, total chemical synthesis of dimeric alkaloids is economically not viable due to their complex structures (Kuboyama et al., 2004; van Der Heijden et al., 2004; Ishikawa et al., 2008). Furthermore, improving the production of monomeric vindoline and dimeric alkaloids in cell suspension and hairy root cultures is challenging as they lack the required level of cellular and tissue differentiation essential for the expression of entire MIAs pathway genes, especially the genes involved in vindoline biosynthesis (Zarate and Verpoorte, 2007; Guirimand et al., 2009). Such a scenario calls for genetic transformation of *C. roseus* for improvement of MIAs production at the whole plant level.

To date, genetic transformation in *C. roseus* has been mostly confined to hairy roots and cell cultures (Choi et al., 2004; Peebles et al., 2006; Jaggi et al., 2011; Sun et al., 2017). Moreover, transgenic *C. roseus* cell suspension cultures do not produce alkaloids in a stable manner and their ability to accumulate MIAs declines by prolonged subculture (Whitmer et al., 2003). Not many efforts have been made with respect to metabolic engineering of *C. roseus* plants, as they are hard-to-transform owing to their highly recalcitrant nature for genetic transformation. Lately, some reports have demonstrated the generation of transgenic *C. roseus* through *Agrobacterium tumefaciens*-mediated transformation (Verma and Mathur, 2011b; Pan et al., 2012; Wang Q. et al., 2012). Direct shoot bud organogenesis and transformation were achieved using pre-plasmolyzed leaf explants and β-glucuronidase (GUS) expression was confirmed in transgenic plants (Verma and Mathur, 2011b). However, there are only few reports pertaining to development of transgenic *C. roseus* overexpressing MIA pathway genes or regulators (Pan et al., 2012; Wang Q. et al., 2012). It was shown that transgenic overexpression of deacetylvindoline-4-*O*-acetyltransferase (*DAT)* in *C. roseus* plants resulted in improved accumulation of vindoline (Wang Q. et al., 2012). Another study from the same group reported enhanced production of ajmalicine, catharanthine and vindoline in transgenic *C. roseus* overexpressing geraniol 10-hydroxylase (*G10H)* and the transcriptional regulator Octadecanoid-derivative Responsive *Catharanthus* AP2-domain (*ORCA3)* (Pan et al., 2012; Wang Q. et al., 2012).

**FIGURE 1:**
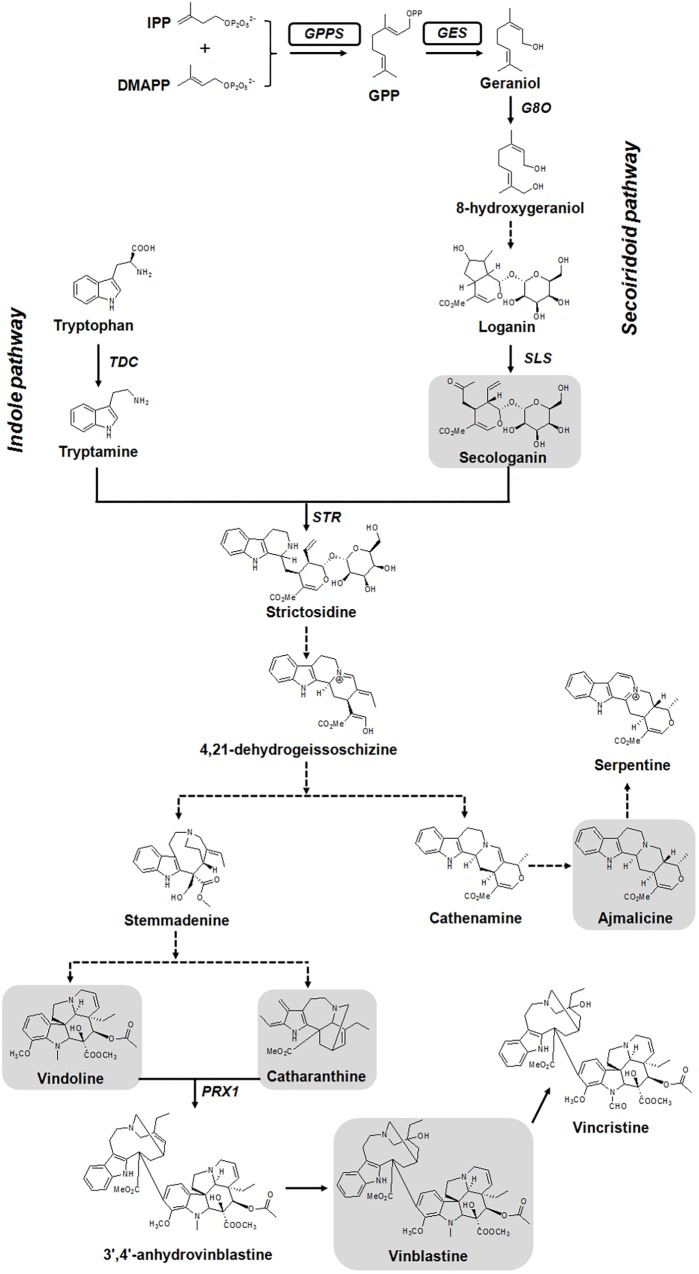
Simplified version of monoterpene indole alkaloids (MIAs) biosynthetic pathway in *C. roseus*. Full and dashed arrows indicate single and multiple enzymatic steps, respectively. The genes used to generate transgenic plants, geranyl(geranyl) diphosphate synthase [*G(G)PPS*] and geraniol synthase (*GES*), are boxed. Structures of analyzed metabolites in this study are shown in gray highlighted boxes. DMAPP, dimethylallyl diphosphate; *G8O*, geraniol-10-hydroxylase/8-oxidase; GPP, geranyl diphosphate; IPP, isopentenyl diphosphate; *PRX1*, peroxidase 1; *SLS*, secologanin synthase; *STR*, strictosidine synthase; *TDC*, tryptophan decarboxylase.

Monoterpene indole alkaloids are derived from the central intermediate strictosidine, which is formed by the condensation of indole pathway derived tryptamine and monoterpene iridoid precursor secologanin in a reaction catalyzed by the enzyme strictosidine synthase (STR) (**Figure 1**). While the indole moiety tryptamine is formed from tryptophan, the iridoid biosynthesis starts from the monoterpene geraniol. Geraniol is formed from geranyl diphosphate (GPP) by the action of a terpene synthase, geraniol synthase (GES). GPP, the universal precursor of monoterpenes, is produced by the condensation of isopentenyl diphosphate (IPP) and dimethylallyl diphosphate (DMAPP) by GPP synthase (GPPS) (Dudareva et al., 2006; Nagegowda, 2010). Geraniol is further oxidized by geraniol-10-hydroxylase/8-oxidase (G8O) to 8-hydroxygeraniol, which is then converted to loganin and finally to secologanin by multiple enzymatic steps (Miettinen et al., 2014). In plants, GPPS functions at the branch point of general and specialized metabolism and regulates flux to monoterpene biosynthesis (Orlova et al., 2009; Nagegowda, 2010). In tobacco, overexpression of snapdragon GPPS small subunit (*GPPS.SSU*) resulted in an increased monoterpene emission in leaves. However, plants were dwarfed and exhibited strong leaf chlorotic symptoms together with increased light sensitivity due to reduction in the level of primary metabolites (Orlova et al., 2009). In another study, co-expression of peppermint *GPS.SSU* with four different monoterpene synthases boosted monoterpene production in transgenic tobacco without negatively affecting plant growth (Yin et al., 2017). These studies indicate that, overexpression of either GPPS alone or co-expression of GPPS with a monoterpene synthase could enhance the production of monoterpenes.

In *C. roseus*, the heteromeric GPPS consisting of an enzymatic large subunit (LSU) and an inactive small subunit (SSU) is involved in providing GPP required for MIA biosynthesis. The LSU is a bifunctional G(G)PP synthase [G(G)PPS] that produces both GPP and GGPP in a ∼2:1 ratio (Rai et al., 2013). Further, GPP is utilized by the monoterpene synthase GES, which is transcriptionally regulated and its transient overexpression in leaves enhanced monomeric alkaloids in *C. roseus* (Kumar et al., 2015). It has been reported in cell cultures that the terpene moiety is limiting for the biosynthesis of MIAs (Peebles et al., 2006). Moreover, we have previously shown by feeding studies that geraniol and not tryptophan is limiting for the biosynthesis of vindoline and catharanthine in *C. roseus* leaves (Kumar et al., 2015). Thus, enhanced availability of early secoiridoid pathway precursors (GPP and geraniol) could push the flux toward secologanin, which in turn could enhance the accumulation of end products (monomeric/dimeric alkaloids). In this work, we overexpressed a bifunctional *G(G)PPS*, and co-expressed *G(G)PPS* and *GES* in transgenic *C. roseus* plants that were generated through *Agrobacterium*-mediated transformation. Transgenic plants accumulated enhanced secologanin, vindoline, catharanthine, ajmalicine and vinblastine without any negative effects on plant growth, thus demonstrating the importance of metabolic engineering at the whole plant level for improvement of monomeric and dimeric MIAs production in *C. roseus*.

## Materials and Methods

### Generation of Plant Overexpression Vectors

For generation of *G(G)PPS* plant overexpression construct, the open reading frame corresponding to 1,152 bp was PCR-amplified from leaf cDNA of *C. roseus* (cv. Dhawal) using full length gene-specific primers (Supplementary Table S1) containing *Xba*I and *Sac*I sites. The amplified coding regions were cloned into the pJET1.2 vector and nucleotide sequences were confirmed by Sanger sequencing. Resulting pJET constructs were restriction digested and *G(G)PPS* was sub-cloned into *Xba*I and *Sac*I sites of pBI121 binary vector under the control of *35S* promoter of Cauliflower mosaic virus (CaMV) to form pBI121::*G(G)PPS* construct (Supplementary Figure S1). *Agrobacterium tumefaciens* strain GV3101 was transformed with pBI121 empty vector and pBI121::*G(G)PPS* construct by freeze-thaw method. For *GES* overexpression, the plant overexpression construct pBI121::*GES* reported in Kumar et al. (2015) was used.

### Transient Overexpression in *C. roseus*

Transient overexpression was performed following the method described in Kumar et al. (2015). Briefly, overnight grown *Agrobacteria* cultures were pelleted and resuspended in infiltration buffer (50 mM MES pH 5.6, 2 mM Na_3_PO_4_, 0.5% glucose and 100 μM acetosyringone) to a final OD_600_ of 0.2. The *Agrobacteria* suspension was incubated at 28°C for 4 h. Infiltration was performed into the first pair of leaves of 3-week-old *C. roseus* plants using a needle-less syringe. Post-infiltration, plants were covered and maintained in the dark for 48 h. Infiltrated leaves were harvested and used for subsequent gene expression and metabolite analyses.

### Generation of Transgenic *C. roseus* Plants

*C. roseus* (cv. Dhawal) seeds were surface sterilized using 4% sodium hypochlorite and inoculated in half strength MS (Murashige and Skoog, 1962) medium. *In vitro* grown hypocotyl and nodal explants were used for generation of *G(G)PPS* and *G(G)PPS*+*GES* transgenic lines, respectively. While *G(G)PPS* transformants were generated by indirect regeneration using hypocotyl explants according to Wang Q. et al. (2012), plants co-expressing *G(G)PPS*+*GES* were generated by direct regeneration using nodal explants following the method reported in Verma and Mathur (2011a).

For generating transgenic plants through indirect regeneration using hypocotyls explants, MS basal medium was supplemented with 250 mg/L proline and 150 mg/L casein hydrolysate (MSCP) with appropriate hormones as follows. Hypocotyl explants were precultured in MSCP1 medium [MSCP with 1.0 mg/L 2,4-dichlorophenoxyacetic acid (2,4-D), 1.0 mg/L α-naphthalene acetic acid (NAA) and 0.1 mg/L zeatin] for 3 days. After co-cultivation with *Agrobacteria* cultures, explants were transferred to MSCP1 selection medium for a week and subsequently transferred to MSCP2 medium [MSCP with 5.0 mg/L 6-benzyl adenine (BA), 0.5 mg/L NAA] for callus induction. Finally, the initiated shoots were transferred to MSCP3 shoot regeneration medium [MSCP with 1.75 mg/L BA, 0.55 mg/L indole-3-acetic acid (IAA)].

For direct regeneration, nodal explants (1–2 cm) were excised from 1-month-old *in vitro* grown *C. roseus* plants. Subsequently, nodal explants were co-cultivated with 1:1 mixture of *Agrobacteria* harboring pBI121::*G(G)PPS* and pBI121::*GES* (OD_600_ 0.4–0.6) for 10 min. The explants were then blot-dried and inoculated into MS-BNT medium containing 1 mg/L BA, 0.1 mg/L NAA and 400 mg/L thiamine HCl. After 3 days of co-cultivation in dark, explants were transferred to fresh MS-BNT medium containing kanamycin and carbenicillin. For shoot proliferation, explants were continued to be cultured in the same medium by subculturing every 10 days. After five rounds of subculturing, well developed shoots were transferred to half strength MS medium for rooting. In both cases, our own modifications were made for rooting as outlined below. After obtaining putative transgenic shoots, they were excised and inoculated initially in rooting media consisting of half strength MS with 2 mg/L indole-3-butyric acid (IBA) and 300 mg/L carbenicillin until root initiation. Later, these plants were transferred to half strength MS with 2.4 μM IBA and 300 mg/L carbenicillin. Plants with well developed roots were transplanted to pots containing 1:1 sterile soilrite:vermicompost mix, and transferred to glass house after acclimation.

### PCR Confirmation of Transgenic Lines and Quantitative Reverse Transcriptase-PCR (qRT-PCR) Analysis

Genomic DNA was isolated from wild type (WT) controls and putative transgenic lines following the protocol reported by Doyle and Doyle (1990). Briefly, leaf disks were ground with preheated extraction buffer (1.4 M NaCl, 20 mM EDTA, 100 mM Tris HCl, 0.2% β-Mercaptoethanol, 2% PVP, and 2% CTAB) and incubated at 65°C for 1 h with intermittent shaking. An equal volume of 24:1 chloroform:isoamyl alcohol was added to separate the aqueous phase. The DNA was precipitated by adding 0.6 volume of ice-cold isopropanol to the aqueous phase and incubated overnight at −20°C. Precipitated genomic DNA was then washed with 70% ethanol and finally resuspended in milli Q water. Transgenic lines were confirmed by PCR screening using appropriate primers. In the case of *G(G)PPS* lines, *35S* promoter-specific forward and *G(G)PPS*-specific reverse primers were used. For screening *G(G)PPS+GES* lines, *35S/nptII* promoter-specific forward and *35S/G(G)PPS/GES/nptII* specific reverse primers were used (Supplementary Table S1). RNA isolation, cDNA synthesis and qRT-PCR were performed in transgenic lines following the procedure reported in Rai et al. (2013) and Kumar et al. (2015). Total RNA was extracted from 100 mg leaf tissue using the Spectrum^TM^ Plant Total RNA kit (Sigma-Aldrich, United States) following manufacturer’s instructions. To remove contaminating genomic DNA, on-column DNase digestion was performed with DNase I (Sigma-Aldrich, St. Louis, MO, United States). Total RNA (2 μg) was used for first strand cDNA synthesis with random hexamer primers using High Capacity cDNA Reverse Transcription kit (Applied Biosystems, United States) as per manufacturer’s instructions. qRT-PCR was performed with a linear range of cDNA using StepOne Real-Time PCR System (Applied Biosystems, United States) and expression of transcripts were normalized using *N227* that was previously reported as an appropriate endogenous gene in *C. roseus* (Pollier et al., 2014). qRT-PCR reaction mixture of 5 μl contained 2.5 μl of 2X Maxima SYBR Green/ROX qPCR master mix (Thermo Scientific, United States), 1:3 diluted cDNA and 2 μM gene-specific primers (Supplementary Table S1). The following conditions were used for qRT-PCR: 94°C for 10 min for first cycle, followed by 94°C for 15 s and 60°C for 15 s for 40 cycles. Fold change differences in gene expression were analyzed by the comparative cycle threshold (*Ct*) method. Relative quantification was carried out by calculating *Ct* to determine fold difference in gene expression [Δ*Ct* target – Δ*Ct* calibrator]. The relative level was determined as 2^−ΔΔCT^. All experiments were repeated using three biological replicates with three technical replicates and data were analyzed statistically as mentioned below.

### Alkaloid Extraction, Analysis, and Quantification

Secologanin, vindoline, catharanthine, ajmalicine, and vinblastine were quantified using High Performance Liquid Chromatography (HPLC) having Photodiode Array (PDA) detector. HPLC (Model: SCL-10AVP, Shimadzu, Japan) equipped with C_18_ symmetry reverse phase column (5 μm, 250 mm × 4.6 mm, Waters, Milford, MA, United States) was used for all analyses. Secologanin was extracted and quantified according to Tikhomiroff and Jolicoeur (2002) with minor modifications. Fresh leaves (50 mg) from 2-month-old plants were extracted in 1.5 ml methanol for 60 min in a sonicating bath. The extract was centrifuged and the supernatant was decolorized by adding activated charcoal. Subsequently, the supernatant was transferred to a fresh tube and evaporated to dryness. The dried residue was dissolved in 20 μl methanol and used for HPLC analysis. Mobile phase for HPLC consisted of 15:85 (v/v) mixture of gradient grade acetonitrile:phosphoric acid (0.1 M, pH 2.0) with a flow-rate of 1.5 ml/min. HPLC was performed in isocratic mode for 20 min.

Vindoline and catharanthine were extracted following protocol of Lourdes Miranda-Ham et al. (2007). Briefly, oven dried leaf tissue (10 mg) collected from 2-month-old plants were grounded in 2 ml methanol and incubated at 55°C for 2 h with occasional shaking. The obtained extract was filtered and evaporated to dryness. The residue was dissolved in 700 μl of 2.5% H_2_SO_4_ and extracted twice with equal volumes of ethyl acetate. Aqueous phase was retained each time and subsequently, pH of the aqueous phase was adjusted to 9.0 using NH_4_OH. Finally, alkaloids were extracted with equal volume of ethyl acetate and the resulting organic phase was evaporated to dryness. The residue containing alkaloids was dissolved in 20 μl methanol and used in HPLC analysis. Mobile phase for HPLC consisted of a mixture of A (0.1 M ammonium acetate buffer, pH 7.3) and B (gradient grade acetonitrile) with 1 ml/min flow rate of 70:30 ratio of A:B for first 5 min of run and then was linearly ramped to 36:64 for next 5 min with flow rate of 1.4 ml/min. Subsequently, the ratio was changed to 20:80 with 1.4 ml/min for next 5 min and finally the flow rate was reduced to 1 ml/min with 70:30 ratios in the last 5 min.

Ajmalicine and vinblastine were extracted and quantified following the method described in Pan et al. (2016). Briefly, oven dried young leaves (50 mg) collected from 2-month-old plants for vinblastine and roots (200 mg) from 1-year-old plants maintained in glass house for ajmalicine were extracted with 1 ml methanol by sonication (30 min). Samples were centrifuged at 12,000 rpm for 10 min at room temperature and the supernatant was decolorized (for leaf samples) by adding activated charcoal. The resulting methanolic extract was evaporated to dryness, resuspended in 20 μl methanol and used for HPLC quantification. The mobile phase consisted of a mixture of 5 mM Na_2_HPO_4_ (pH 6.0) (solvent A) and gradient grade methanol (solvent B) at a flow rate of 1.5 ml per min. The change in mobile phase was set to a linear gradient from 86:14 to 14:86 at 0–26 min in isocratic mode (14:86 v/v at 26–30 min), a linear gradient from 14:86 to 86:14 at 30–35 min, an isocratic elution with 14:86 (v/v) at 35–40 min. Data was extracted at 238 nm for secologanin, 254 nm for vindoline, catharanthine, ajmalicine, and vinblastine. Peak area obtained from authentic standards (Sigma-Aldrich, St. Louis, MO, United States) (Supplementary Figure S2) and samples were used to quantify alkaloids and expressed as relative content in %.

### Chlorophyll and Carotenoids Quantification, and Phenotypic Analyses

The amount of total chlorophyll and carotenoids was quantified according to Lichtenthaler (1987). Briefly, 20 mg of leaf (third developmental stage) was extracted in 98% ethanol for 2 h. The supernatant was used for quantification of chlorophyll and carotenoids. The absorbance was measured at 470, 653, and 666 nm and the amount of chlorophyll a, chlorophyll b, and total carotenoids was calculated. Flowers of T_0_ transgenic lines were selfed individually and siliques were collected separately from individual transgenic lines. The seeds were germinated and screened by PCR to select the first generation T_1_ positive plants from each transgenic line. Phenotypic parameters such as number of leaves, branches, flowers and siliques formed in WT and selected T_1_ transgenic lines were analyzed.

### Copy Number Determination in Transgenic *C. roseus* Lines

Copy number of *G(G)PPS* and *GES* in transgenic *G(G)PPS* and *G(G)PPS*+*GES* lines were confirmed by using qPCR as described previously (Weng et al., 2004; Yi et al., 2008; Li et al., 2017). For absolute quantification, standard curve was generated using twofold dilutions of genomic DNA extracted from WT controls, selected *G(G)PPS* and all three lines of *G(G)PPS+GES* using primers of *G(G)PPS*, *GES*, and tryptophan decarboxylase [*TDC*, the endogenous single copy gene as determined by Goddijn et al. (1994)]. Three separate sets of reactions were performed to amplify *G(G)PPS*, *GES*, and *TDC*. Each reaction (5 μl) contained 2.5 μl of 2X Maxima SYBR Green PCR master mix (Thermo Scientific, United States), 100 ng genomic DNA and 2 μM gene-specific primers. Reaction was performed as follows: 94°C for 10 min for first cycle, followed by 94°C for 15 s and 60°C for 15 s for 40 cycles. The PCR efficiency of target genes and *TDC* were calculated from slope of standard curve for each gene. The number of copies of *G(G)PPS* and *GES* were determined according to Pfaffl (2001).

### Statistical Analysis

Average mean, standard error (SE) and number of replicates (*n*) used for individual experiment were employed for statistical analysis using the GraphPad QUICKCALC online software^1^. Statistical significance of differences between control and samples were tested according to the unpaired Student’s *t*-test.

## Results

### Transient Overexpression of *G(G)PPS* and *G(G)PPS+GES* Resulted in Increased Alkaloid Accumulation

**FIGURE 2:**
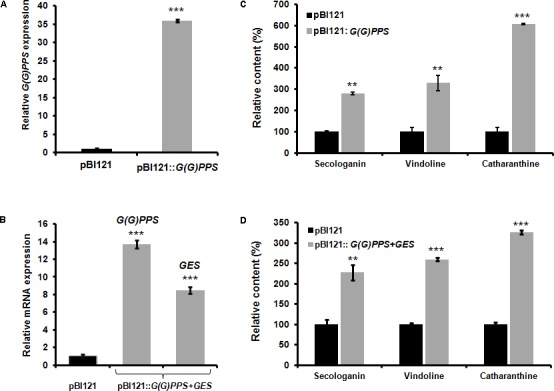
Effect of transient overexpression of *G(G)PPS* and *GES* in *C. roseus* leaves. mRNA expression **(A,B)** and metabolite **(C,D)** analyses in *C. roseus* leaves infiltrated with *A. tumefaciens* carrying pBI121 empty vector (black bar), pBI121::*G(G)PPS* and co-infiltrated with pBI121::*G(G)PPS*+pBI121::*GES* (gray bar) constructs. Transcripts were analyzed by qRT-PCR with *CrN227* as an endogenous reference gene. Expression levels were normalized to *CrN227* and are represented as expression relative to the pBI121 controls that was set to 1. Relative amounts of secologanin, vindoline, and catharanthine in *C. roseus* leaves were quantified by HPLC analysis. Secologanin was extracted from 50 mg (fresh weight) of leaves and quantified following Tikhomiroff and Jolicoeur (2002). The monomeric alkaloids vindoline and catharanthine were extracted using 10 mg of oven dried leaves according to Lourdes Miranda-Ham et al. (2007), and quantified following Kumar et al. (2015). In all cases, first pair of infiltrated leaves were used for alkaloid extraction and quantified by HPLC. The levels of quantified metabolites are expressed in % relative to pBI121 vector infiltrated leaves. The bars represent mean ± standard error (SE) of three independent experiments. Significant differences at *P* < 0.01 and *P* < 0.001 are represented by “^∗∗^” and “^∗∗∗^”, respectively.

Transient overexpression by agroinfiltration either in the host plant or in heterologous systems has been widely used for determining the gene function. Moreover, it is an efficient strategy for functional analyses of genes in those plants where the study of gene function is limited owing to very low transformation efficiency and recalcitrant nature of the plant for genetic transformation. Hence, before proceeding for genetic transformation of *C. roseus* with *G(G)PPS* and *G(G)PPS+GES*, we carried out transient overexpression of these genes to determine their effect on MIA biosynthesis. As *G(G)PPS* and *GES* were previously reported to positively influence MIA biosynthesis in *C. roseus* (Rai et al., 2013; Kumar et al., 2015), we hypothesized that improved availability of precursors (GPP and geraniol) by overexpression of *G(G)PPS* and co-expression of *G(G)PPS+GES* could enhance MIA accumulation in *C. roseus* leaves. As the MIA content varies with leaf developmental stages, both genes were transiently overexpressed in first pair of leaves (that exhibit highest alkaloid accumulation). While, overexpression of *G(G)PPS* resulted in >30-fold transcript increase, co-expression of *G(G)PPS*+*GES* lead to ∼14- and 8-fold increase in transcripts of *G(G)PPS* and *GES*, respectively (**Figures 2A,B**). Next, to determine the effect of *G(G)PPS* and *G(G)PPS+GES* overexpression on alkaloid accumulation, first we checked the level of terpenoid intermediate secologanin. HPLC analysis showed ∼2.8- and 2.2-fold increase in secologanin levels in *G(G)PPS* and *G(G)PPS+GES* infiltrated leaves, respectively, compared to empty vector pBI121 controls (**Figures 2C,D** and Supplementary Figures S3A,B). Subsequent analysis of the monomeric alkaloids in *G(G)PPS* infiltrated leaves revealed ∼3- and ∼6-fold increase in vindoline and catharanthine, respectively (**Figure 2C** and Supplementary Figure S3A). Similarly, *G(G)PPS+GES* co-infiltrated leaves exhibited an increase of ∼2.5 and ∼3.2-fold vindoline and catharanthine (**Figure 2D** and Supplementary Figure S3B). Higher accumulation of secologanin, vindoline and catharanthine could be attributed to dramatic increase in the availability of accumulated transcripts as a result of transient overexpression. Taken together, our results clearly demonstrated that overexpression of *G(G)PPS* and *G(G)PPS+GES* can improve the metabolic flux toward MIA biosynthesis in *C. roseus.*

### Generation of Transgenic *C. roseus* Plants Overexpressing *G(G)PPS* and *G(G)PPS+GES*

**FIGURE 3:**
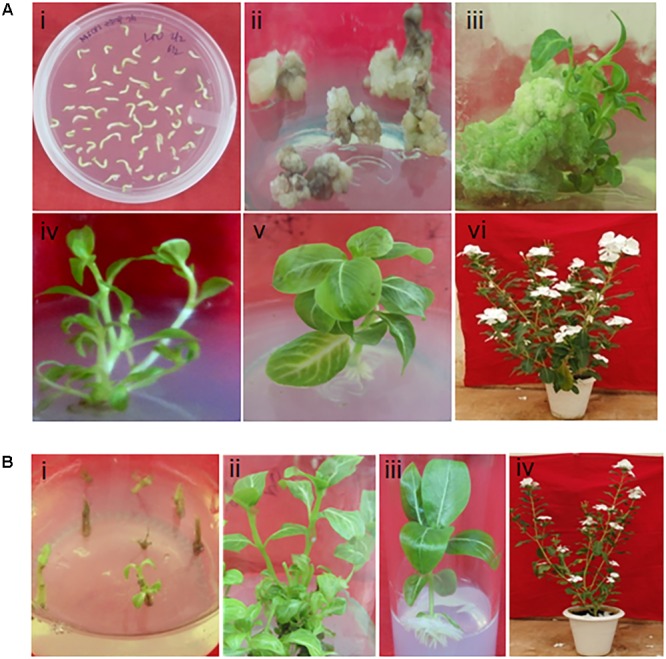
Generation of transgenic *C. roseus* plants overexpressing *G(G)PPS* and *GES*. **(A)** Transformation of *C. roseus* using hypocotyl explants for *G(G)PPS* overexpression. **(i)** Preculture of hypocotyl explants in MS basal media supplemented with 250 mg/L proline and 150 mg/L casein hydrolysate (MSCP) media; **(ii)** callus induction in MSCP1 media supplemented with 40 mg/L kanamycin and 300 mg/L carbenicillin; **(iii)** callus proliferation and shoot initiation in MSCP2 with 70 mg/L kanamycin and 300 mg/L carbenicillin; **(iv)** shoot elongation in MSCP3 with 90 mg/L kanamycin and 300 mg/L carbenicillin; **(v)** root proliferation in half strength MS media with 2.4 μM IBA; **(vi)** Established transgenic *G(G)PPS* plant in the flowering stage. **(B)** Transformation of *C. roseus* nodal explants for *G(G)PPS*+*GES* overexpression. (**i** and **ii**) Selection of nodal explants and multiple shoot induction in MS media supplemented with 1 mg/L BAP + 0.1 mg/L NAA + 400 mg/L thiamine HCl + 70 or 100 mg/L kanamycin + 300 mg/L carbenicillin, respectively; **(iii)** root proliferation in half strength MS media with 2.4 μM IBA; **(iv)** Established transgenic *G(G)PPS+GES* plant in the flowering stage.

In order to substantiate our findings of transient overexpression studies and to demonstrate the *in planta* role of *G(G)PPS* and *G(G)PPS+GES* in improving MIA production, we generated transgenic *C. roseus* overexpressing *G(G)PPS* and *G(G)PPS+GES.* So far, very few efforts have been made with respect to metabolic engineering of *C. roseus* plant due to its highly recalcitrant nature for genetic transformation together with low transformation efficiency. Lately, some reports have shown the generation of transgenic *C. roseus* through *A. tumefaciens*-mediated transformation. For the generation of transgenic *C. roseus* plants, culture conditions were optimized for callus induction and indirect regeneration following Wang Q. et al. (2012). *In vitro* grown hypocotyls were used as explants for *Agrobacterium*-mediated genetic transformation. Hypocotyl explants were precultured in MSCP1 (**Figure 3Ai**) for 3 days prior to *Agrobacterium* infection as it has been reported to improve the rate of transformation in *C. roseus* (Alam et al., 2017). After co-cultivation with *Agrobacterium-*harboring overexpression constructs, callus initiation was observed in most of the explants in a week in MSCP1 supplemented with 40 mg/L kanamycin and 300 mg/L carbenicillin. The cream colored friable calli developed after about 3 weeks were sub-cultured on to MSCP2 selection medium (**Figure 3Aii**). However, the calli upon subculturing in MSCP2 medium turned brown in most of the cases and died in about 1–2 weeks. After 2 weeks of sub-culturing in MSCP2 selection medium, only 2–3% of survived calli turned into green friable calli and shoot initiation was observed (**Figure 3Aiii**). Further, the initiated shoots were transferred to MSCP3 regeneration medium containing appropriate antibiotics. Although the regeneration efficiency was very less, multiple shoots were induced from embryogenic calli in some of the transformation events (**Figure 3Aiv**). From each calli, 3–4 shoots were induced and after 1 month of shoot maturation, healthy shoots were transferred initially to half strength MS medium supplemented with 2 mg/L IBA. After 2 weeks, though shoots exhibited root initiation, leaves turned yellow and exhibited necrotic symptoms in most of the cases. Hence, a lower concentration of IBA (2.4 μM) was used for root proliferation according to Dhandapani et al. (2008) (**Figure 3Av**). Plants with well developed roots were transferred to 1:1 sterile soilrite:vermicompost mix and maintained in glass house conditions (**Figure 3Avi** and Supplementary Figures S4A,B). The complete process of transformation using hypocotyl explants from seed germination to hardening of putative transgenic lines took approximately 4 months. Regeneration efficiency from hypocotyl explants was very low as mentioned above and we were able to generate seven *C. roseus* lines overexpressing *G(G)PPS*. For PCR screening of putative transformants, *35S* forward and two different *G(G)PPS* specific reverse primers were used. In both primer combinations, seven plants out of 18 kanamycin-selected plants were positive for *G(G)PPS* confirming the transgenic nature (Supplementary Figure S5A).

The regeneration efficiency from embryogenic calli was found to be low (3%) and there was no regeneration in the case of explants co-transformed with *G(G)PPS*+*GES.* Hence, an alternate approach of genetic transformation using nodal explants was followed for co-transformation. After co-cultivation with *Agrobacteria* harboring *G(G)PPS* and *GES* overexpression constructs, nodal explants were selected in MS-BNT medium supplemented with 70 mg/L kanamycin and 300 mg/L carbenicillin (**Figure 3Bi**). Most of the co-cultivated explants turned brown and died upon transferring to selection media. Healthy explants were sub-cultured for five more rounds in kanamycin selection media with 70 mg/L. Subsequently, the kanamycin concentration was increased to 100 mg/L for next two rounds. The explants exhibited initiation of leaves and shoot elongation from third round of selection (∼1 month after co-cultivation). In most of the survived explants after five rounds of antibiotic selection, shoot bud induction as well as proliferation occurred from nodes resulting in 3–7 multiple shoots (**Figure 3Bii**). Individual shoots were then transferred to fresh selection media every 15 days and healthy surviving shoots were inoculated into rooting medium. Although root initiation was observed in rooting media containing 2 mg/L IBA, root proliferation was only obtained in a much lower IBA (2.4 μM) concentration (**Figure 3Biii**). Well rooted plants were transferred to pots containing 1:1 sterile soilrite:vermicompost mix (**Figure 3Biv** and Supplementary Figures S4A,B). After obtaining putative transformants of *G(G)PPS+GES*, PCR analysis using genomic DNA isolated from leaves was carried out to identify transgenic nature of plants. Screening of *G(G)PPS*+*GES* lines was carried out by using *nptII* forward and reverse primers, and *35S* forward and gene specific [(*G(G)PPS* or *GES*] reverse primers. PCR results indicated three plants positive for *G(G)PPS+GES* out of 16 kanamycin selected plants (Supplementary Figures S5B,C). The transformation efficiency using nodal explants was also ∼3% similar to transformation using hypocotyls explants. PCR analysis using genomic DNA with primers specific for *Agrobacterium* chromosomal virulence gene (*ChvA*) revealed the absence of the corresponding band, thus ruling out the possibility of *Agrobacterium* contamination in both *G(G)PPS* and *G(G)PPS+GES* transformants (Supplementary Figure S6).

### Ectopic Expression of *G(G)PPS* and *G(G)PPS+GES* Elevates Alkaloid Accumulation in *C. roseus* Transgenic Plants

**FIGURE 4:**
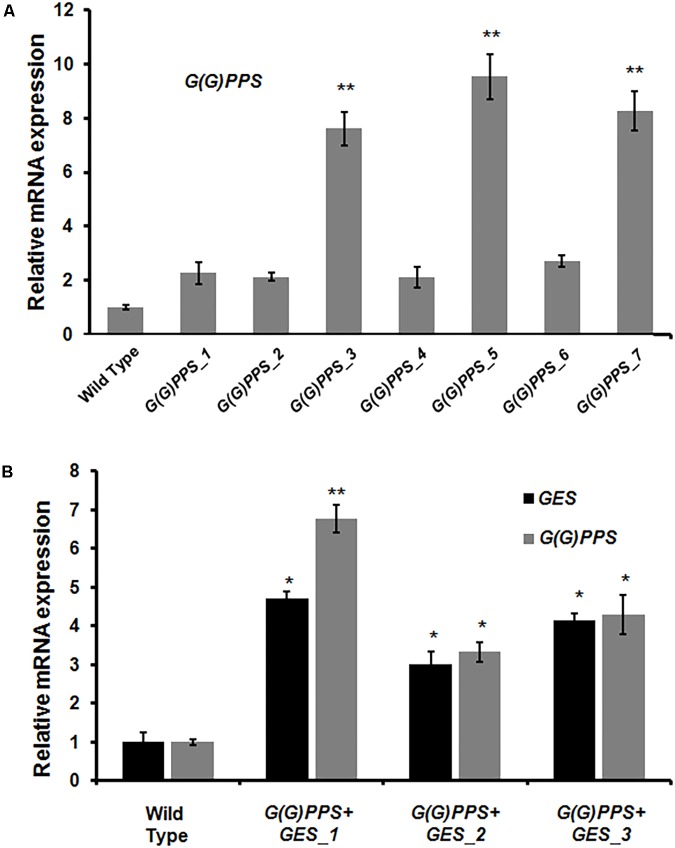
Analysis of gene expression in transgenic *G(G)PPS* and *GES C. roseus* plants. qRT-PCR analysis of *G(G)PPS* (gray bar) **(A)** and *G(G)PPS* (gray bar)/*GES* (black bar) **(B)** in transgenic *C. roseus* plants. Expression levels of genes were normalized to the endogenous reference gene *CrN227* and are represented relative to the wild type (WT) controls, which was set to 1. Error bars represent mean ± standard error (SE) of three independent experiments. Significant differences at *P* < 0.05 and *P* < 0.01 are represented by “^∗^” and “^∗∗^”, respectively.

**FIGURE 5:**
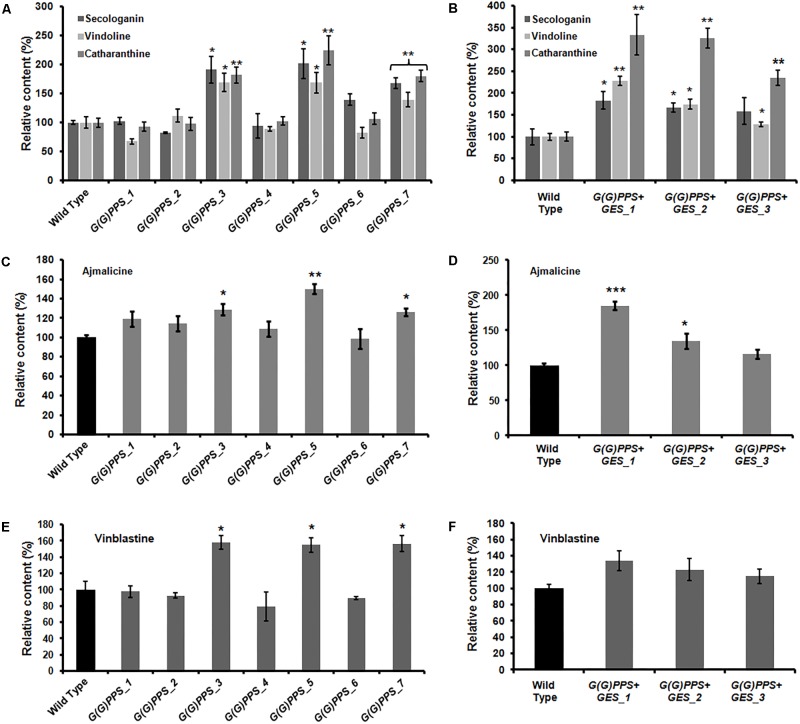
Quantification of metabolites in the T_0_ lines of transgenic *G(G)PPS* and *GES C. roseus* plants. Relative amounts of secologanin, vindoline and catharanthine **(A,B)**, ajmalicine **(C,D)**, and vinblastine **(E,F)** in transgenic *G(G)PPS* and *G(G)PPS+GES C. roseus* lines. Secologanin was extracted from 50 mg (fresh weight) of leaves and quantified following Tikhomiroff and Jolicoeur (2002). The monomeric alkaloids vindoline and catharanthine were extracted using 10 mg of oven dried leaves according to Lourdes Miranda-Ham et al. (2007), and quantified following Kumar et al. (2015). Ajmalicine was extracted from roots of wild type (WT) and transgenic plants using 200 mg fresh weight tissue following Pan et al. (2016). The methanolic extract was subjected to HPLC analysis to quantify ajmalicine. The dimeric alkaloid vinblastine was extracted using 50 mg of oven dried leaves and quantified according to Pan et al. (2016). In all cases, young leaves or roots of same developmental stages were used for alkaloid extraction and were quantified by using HPLC. The quantified metabolites are expressed as % levels relative to WT controls. Error bars represent mean ± standard error (SE) of three to four independent experiments. Significant differences at *P* < 0.05, *P* < 0.01, and *P* < 0.001 are represented by “^∗^”, “^∗∗^”, and “^∗∗∗^”, respectively.

Having confirmed the transgenic nature of *C. roseus* plants, the transcript abundance of *G(G)PPS* and *GES* in transgenic lines was analyzed by qRT-PCR. First, the transcript levels in all transgenic lines were determined with respect to WT control plants that were also generated through tissue culture. The various transgenic lines showed increased expression of *G(G)PPS* with a 2- to 9.5-fold increase compared to WT control plants, with maximum expression in *G(G)PPS_5* followed by 8-fold in *G(G)PPS_3* and *G(G)PPS_7* (**Figure 4A**). Transgenic plants co-expressing *G(G)PPS+GES* showed increased expression of both genes (**Figure 4B**). The expression of *G(G)PPS* was ∼7-, 3-, and 4-fold in lines 1, 2 and 3, respectively, whereas the expression of *GES* was ∼5-fold in line 1, and ∼3-fold in lines 2 and 3 (**Figure 4B**). Next, to determine whether the changes in gene expression were also accompanied by increased MIA accumulation, metabolites were extracted from independent transgenic lines and quantified by HPLC. The content of terpenoid intermediate secologanin exhibited a significant boost of ∼2-fold in lines *G(G)PPS_3*, *G(G)PPS_5* and *G(G)PPS_7* that also showed highest transcript abundance (**Figure 5A** and Supplementary Figure S7A). There was no significant enhancement of secologanin in the remaining four *G(G)PPS* lines that correlated with the gene expression of *G(G)PPS* (**Figure 5A**). Further, to check whether improved availability of secologanin resulted in enhanced accumulation of monomeric alkaloids in leaves, vindoline and catharanthine levels were analyzed in *G(G)PPS* transgenic lines. HPLC analysis revealed that the trend in accumulation of monomers correlated with the level of secologanin in all seven lines with significant increase of 1.5- to 1.7-fold vindoline and 1.7- to 2.0-fold catharanthine in lines *G(G)PPS*_3, *G(G)PPS*_5 and *G(G)PPS*_7 (**Figure 5A** and Supplementary Figure S7A). Similarly, *G(G)PPS*+*GES* co-expressing lines exhibited a significant increase in secologanin (∼1.8-fold) along with significant increase in vindoline (1.6- to 2.2-fold) and catharanthine (2.5- to 3.0-fold) (**Figure 5B** and Supplementary Figure S7B). Although all three *G(G)PPS*+*GES* lines showed significant accumulation of secologanin and vindoline similar to *G(G)PPS* lines, the level of catharanthine was much higher (>3.0-fold) in co-expressing lines (**Figure 5B** and Supplementary Figure S7B).

In MIA biosynthesis, 4,21-dehydrogeissoschizine acts as a branch point intermediate for the formation of ajmalicine and stemmadenine. While ajmalicine is converted to serpentine, stemmadenine undergoes several transformations to form catharanthine and vindoline (Peebles et al., 2011). In order to assess the impact of *G(G)PPS* and *GES* overexpression on accumulation of root alkaloid, ajmalicine was quantified in roots of WT controls and transgenic lines by HPLC analysis. Consistent with the effect on secologanin, catharanthine and vindoline levels, *G(G)PPS* and *GES* overexpression enhanced the level of ajmalicine by ∼1.25- to 1.5-fold in three high expressing *G(G)PPS* transgenic lines (**Figure 5C**) and 1.3- to 1.8-fold in *G(G)PPS+GES* co-expressing lines (**Figure 5D**). This indicated that improved availability of GPP and geraniol due to overexpression of *G(G)PPS* and *GES* also influenced the accumulation of alkaloids in roots.

### Differential Expression of Peroxidise 1 in Transgenic Lines Correlated With Vinblastine Accumulation

**FIGURE 6:**
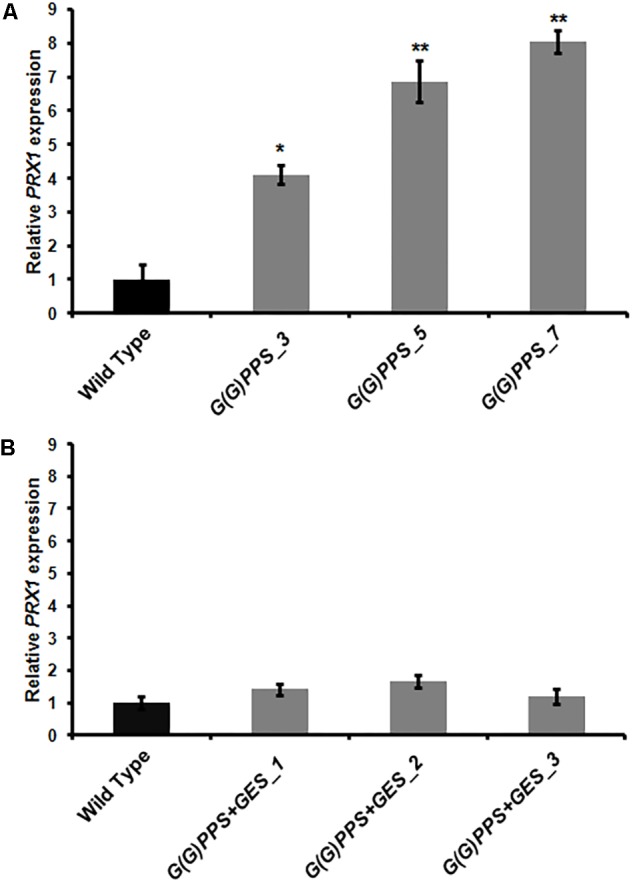
*PRX1* expression in transgenic *C. roseus* plants. Analysis of *PRX1* expression by qRT-PCR in *G(G)PPS* **(A)** and *G(G)PPS*+ *GES* **(B)** overexpressing transgenic *C. roseus* plants. Expression levels of *PRX1* were normalized to the endogenous reference gene *CrN227* and are represented relative to the WT controls, which was set to 1. Error bars represent mean ± standard error (SE) of three independent experiments. Significant differences at *P* < 0.05 and *P* < 0.01 are represented by “^∗^” and “^∗∗^”, respectively.

A significant increase of vinblastine (∼1.5-fold) was observed in three *G(G)PPS* overexpressing lines that exhibited increased secologanin, vindoline and catharanthine (**Figures 5A,E**). With respect to alkaloid accumulation in *G(G)PPS*+*GES* co-expressing plants, although there was elevated accumulation of terpenoid intermediate secologanin, and monomeric alkaloids, there was no significant increase in the level of vinblastine in any of the lines (**Figures 5B,F**). In a similar manner, when *ORCA3* was expressed alone or co-expressed with *G10H* in transgenic *C. roseus* plants, monomers were significantly enhanced without any effect on accumulation of α-3′,4′-anhydrovinblastine and vinblastine (Pan et al., 2012). Peroxidise 1 (PRX1) mediates the coupling of monomeric catharanthine and vindoline into dimeric α-3′,4′-anhydrovinblastine, the immediate precursor for vinblastine (Costa et al., 2008). The fact that, the vinblastine level was boosted only in *G(G)PPS* transgenic plants and not in *G(G)PPS*+*GES* lines indicated a possible differential regulation at the terminal step involving PRX1. To verify this, transcript levels of *PRX1* were determined in *G(G)PPS* and *G(G)PPS*+*GES* transgenic *C. roseus* lines. The analyses revealed a significant difference of *PRX1* transcripts levels in *G(G)PPS* and *G(G)PPS*+*GES* transgenic lines (**Figure 6**). All three higher alkaloid accumulating *G(G)PPS* lines [*G(G)PPS*_3, *G(G)PPS*_5, and *G(G)PPS*_7] showed enhanced *PRX1* transcript abundance ranging from fourfold to eightfold (**Figure 6A**). However, *G(G)PPS+GES* co-expressing lines exhibited *PRX1* expression similar to that of WT controls (**Figure 6B**). This differential expression of *PRX1* could be the possible reason for contrasting levels of vinblastine in *G(G)PPS* and *G(G)PPS*+*GES* transgenic plants.

### Transgenic *C. roseus* Plants Exhibited Stable Levels of Gene Expression and Alkaloids in the T_1_ Generation

**FIGURE 7:**
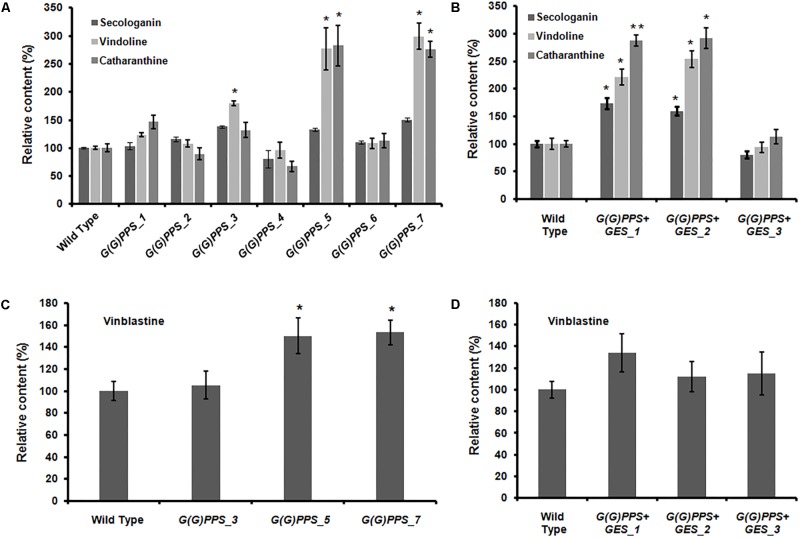
Analyses of metabolites in T_1_ lines of transgenic *G(G)PPS* and *G(G)PPS+ GES* in *C. roseus* plants. Relative levels of secologanin, vindoline and catharanthine **(A,B)**, and vinblastine **(C,D)** in transgenic *G(G)PPS* **(A,C)** and *G(G)PPS+GES* **(B,D)** *C. roseus* lines. In the case of *G(G)PPS* transgenic plants, only those lines showing significant increase in the monomeric alkaloids catharanthine and vindoline **(A)** were considered for the dimeric vinblastine analysis **(B)**. Error bars represent mean ± standard error (SE) of three to four independent experiments. Significant differences at *P* < 0.05 and *P* < 0.01 are represented by “^∗^” and “^∗∗^”, respectively.

To determine the genetic and chemostability, we checked the transcript and metabolite levels in PCR positive T_1_ lines of *G(G)PPS* and *G(G)PPS+GES* (**Figure 7** and Supplementary Figures S8, S9). First, transgene copy numbers in selected *G(G)PPS* [*G(G)PPS*_1, *G(G)PPS*_3, *G(G)PPS*_5, and *G(G)PPS*_7] and *G(G)PPS+GES* transgenic lines were determined by qPCR using genomic DNA extracted from WT controls and transgenic lines. The correlation coefficients of the standard curves of target genes, *G(G)PPS, GES*, and *TDC* internal control were 0.996–0.998, and PCR efficiencies were close to 97–100%. This indicated the accuracy and robustness in estimating copy number of genes based on the standard curves. The analysis revealed that *G(G)PPS_1, G(G)PPS*_3, and *G(G)PPS*_7 showed the presence of two copies, whereas line *G(G)PPS*_5 exhibited the presence of three copies of *G(G)PPS* (Supplementary Table S2). With respect to co-expressing lines, *G(G)PPS+GES_*1 showed three copies, whereas lines *G(G)PPS+GES_2* and *G(G)PPS+GES_3* possessed two copies of *G(G)PPS*. The copy number of *GES* was found to be three in all the co-expressing transgenic lines (Supplementary Table S2). The WT controls showed one copy each for endogenous *G(G)PPS* and *GES*. Hence, the determined copy numbers also include the endogenous copy of *G(G)PPS* and *GES* in all transgenic events.

Transcript levels in the T_1_ *G(G)PPS* transgenic plants exhibited higher *G(G)PPS* expression (8- to 10-fold) in *G(G)PPS_5* and *G(G)PPS_7* lines (Supplementary Figure S9A) similar to the levels observed in the T_0_ lines. However, *G(G)PPS_3* line that showed significantly higher transcript accumulation (∼8-fold) in the T_0_ generation, exhibited reduced expression in the T_1_ generation was comparable to WT control plants (**Figure 4A** and Supplementary Figure S9A). In co-transformed *C. roseus* lines, *G(G)PPS* transcript abundance was sixfold in line *G(G)PPS+GES_1* and ∼3-fold in other two lines (Supplementary Figure S9B), whereas the expression of *GES* varied between threefold and fourfold in all three lines (Supplementary Figure S9B). To confirm whether increased gene expression was accompanied by elevated MIA accumulation in transgenic lines, alkaloids were quantified in T_1_ *G(G)PPS* and *G(G)PPS+GES* lines. Similar to the accumulation observed in the T_0_ generation, T_1_ *G(G)PPS* lines exhibited improved accumulation of monomeric alkaloids with no significant change in levels of secologanin (**Figure 7A** and Supplementary Figure S10A). While *G(G)PPS_5* and *G(G)PPS_7* exhibited ∼2.5-to 2.7-fold accumulation of vindoline and catharanthine, *G(G)PPS_3* showed significant increase of only vindoline (∼1.8-fold) (**Figure 7A**). As for the dimeric alkaloids, only *G(G)PPS*_5 and *G(G)PPS*_7 exhibited ∼1.5-fold increase in vinblastine compared to WT controls (**Figure 7C** and Supplementary Figure S10A). Among the co-expressing *G(G)PPS+GES* plants, only two T_1_ plants showed improved accumulation of secologanin, (∼1.5-fold), vindoline (∼2.2-fold), and catharanthine (∼2.8-fold) (**Figure 7B** and Supplementary Figure S10B). Also, similar to the T_0_ plants, there was no increase in the level of vinblastine in any of the T_1_ *G(G)PPS+GES* co-expressing lines possibly due to regulation of *PRX1* (**Figure 7D** and Supplementary Figure S10B).

## Discussion

Vinblastine and vincristine are blockbuster anti-cancer drugs extracted exclusively from *C. roseus* leaves. As these dimeric MIAs are accumulated at very low levels in leaves, increasing their foliar concentrations would reduce the cost of production. Hence, this study was taken up to enhance the production of monomeric and dimeric MIAs by overexpression of early secoiridoid pathway genes in transgenic *C. roseus* plants. Previous efforts of overexpression or silencing of transcriptional regulators and pathway genes in *C. roseus* cell cultures have had limited success in improving MIAs production, indicating a complex regulatory mechanism that balances metabolic flux and thus MIAs accumulation in *C. roseus* (Murata and De Luca, 2005; Peebles et al., 2009; Jaggi et al., 2011; Van Moerkercke et al., 2015; Sun et al., 2017). In order to allow higher metabolic outputs, improving the availability of upstream precursors is an alternative strategy. Initial elicitation and feeding studies using indole and terpenoid precursors in *C. roseus* cell cultures indicated inconsistent results on accumulation of different MIAs including serpentine, ajmalicine, tabersonine, and strictosidine (Moreno et al., 1993; Whitmer et al., 1998; Contin et al., 1999). However, later studies in cell suspension and hairy roots of *C. roseus* indicated that the terpenoid moiety, geraniol (not indole moiety, tryptophan) has a positive effect on accumulation of MIAs like tabersonine and ajmalicine (Morgan and Shanks, 2000; Lee-Parsons and Royce, 2006). In addition, we have previously shown at the whole plant level that GPPS and GES play important roles in providing metabolic flux toward downstream MIA biosynthesis in *C. roseus* (Rai et al., 2013; Kumar et al., 2015). Since GPPS and GES provide starting precursors (GPP and geraniol) for the terpene moiety formation, we hypothesized that overexpression of *G(G)PPS* and co-expression of *G(G)PPS+GES* could push the flux toward secologanin biosynthesis ultimately leading to improved MIA production. To this end, we performed transient overexpression of these genes by agroinfiltration as this strategy has been used to validate gene function in different medicinal plants including *C. roseus* (Raina et al., 2012; Rai et al., 2013; Kumar et al., 2015). Transient overexpression of *G(G)PPS* and *G(G)PPS*+*GES* lead to enhanced secologanin and the monomeric MIAs (**Figure 2** and Supplementary Figure S3), indicating that stable overexpression of these genes in transgenic *C. roseus* plants could result in improved production of MIAs.

The lack of a reliable genetic transformation method in *C. roseus* at the whole plant level has been a major impediment for improving MIAs by metabolic engineering approaches (Zarate and Verpoorte, 2007). Owing to the recalcitrant nature and low transformation efficiency, only a few reports have demonstrated genetic transformation in *C. roseus* using genes related to MIA biosynthesis (Pan et al., 2012; Wang Q. et al., 2012). Here, we generated transgenic *C. roseus* overexpressing *G(G)PPS* using transformation protocol of Wang Q. et al. (2012) with minor modifications. Although Wang Q. et al. (2012) reported transformation efficiency of 11% for transgenic *C. roseus* expressing *DAT* gene, in our case the efficiency of transformation was ∼3%. As hypocotyl explants co-cultivated with *G(G)PPS*+*GES* constructs failed to generate shoots, an alternate regeneration method using nodal explants as reported by Verma and Mathur (2011a) was employed for transformation. This strategy yielded only three transformants with ∼3% transformation efficiency. The lower transformation efficiency in this study could be due to the genotype of *C. roseus* used as it has been shown in other plant species that transformation efficiency depends on the genotype and cultivar (Wang Y. et al., 2012; Udayakumar et al., 2014). Transgenic *G(G)PPS* and *G(G)PPS+GES* lines exhibited varied levels of transcript abundance for corresponding genes (**Figure 4**). *G(G)PPS* expression ranged from 2- to 9.5-fold and 3- to 6-fold in *G(G)PPS* and *G(G)PPS*+*GES* transgenic lines, respectively, whereas *GES* expression was 3- to 4.5-fold in co-expressing lines (**Figure 4**). Similar to our observation, overexpression of *DAT* in transgenic *C. roseus* resulted in twofold to sevenfold expression in different lines (Wang Q. et al., 2012). In another report, overexpression of transcriptional regulator *ORCA3* alone in transgenic *C. roseus* plants resulted only in ∼2-fold expression, whereas transgenic lines co-expressing *G10H* and *ORCA3* resulted in ∼5- and 7-fold increase in transcript levels of *G10H* and *ORCA3*, respectively (Pan et al., 2012). Increased transcript accumulation of *G(G)PPS* and *G(G)PPS*+*GES* in transgenic *C. roseus* plants enhanced the content of secoiridoid secologanin, monomeric vindoline and catharanthine, and root alkaloid ajmalicine (**Figures 5** and Supplementary Figure S7). Overall, the level of secologanin and vindoline in three higher expressing *G(G)PPS* lines and in all *G(G)PPS*+*GES* transgenic lines remained similar (**Figures 5A,B**). However, there was higher accumulation of catharanthine in two *G(G)PPS*+*GES* lines compared to *G(G)PPS* lines (**Figures 5A,B**), suggesting that improved availability of both GPP and geraniol could have enhanced the accumulation of catharanthine. It was reported that co-expression of 1-deoxy-D-xylulose-5-phosphate synthase (*DXS*) and *G10H* in hairy roots resulted in improved accumulation of ajmalicine and tabersonine compared to expression of *DXS* alone (Peebles et al., 2011). Similarly, *C. roseus* transgenic plants co-expressing *ORCA3* and *G10H* exhibited higher production of ajmalicine compared to plants expressing *ORCA3* alone (Pan et al., 2012). These prior findings along with our present results suggest that overexpression of multiple genes may be necessary to achieve significant gains in product accumulation especially in pathways involving multiple branches, where precursors are channeled into diverse metabolites (Peebles et al., 2011).

In addition to enhancing secologanin and monomeric alkaloids, *G(G)PPS* overexpressing lines also accumulated higher level of vinblastine (**Figure 5E** and Supplementary Figure S7A). In contrast, *G(G)PPS*+*GES* lines did not show any increase in the levels of vinblastine despite of significant increase in secologanin, vindoline, and catharanthine (**Figure 5F**). This result suggested that improved metabolic flux (in the form of monomeric alkaloids) is somehow not being utilized for dimeric alkaloid formation. These findings prompted us to check whether gene expression of *PRX1* has any influence on the accumulation of vinblastine. Transcript analysis revealed that *G(G)PPS* and *G(G)PPS*+*GES* overexpressors had a differential expression of *PRX1* that correlated with the accumulation of vinblastine (**Figures 5E,F** and Supplementary Figure S7). It is not clear at this point, how *PRX1* is differentially expressed in *G(G)PPS* and *G(G)PPS*+*GES* overexpressors. However, *PRX1* may be transcriptionally regulated by some unknown mechanism in *G(G)PPS* overexpressors leading to enhanced vinblastine. It has been previously reported that the expression of *PRX1* and accumulation of 3′,4′-anhydrovinblastine is under complex regulation and also depends on the availability of hydrogen peroxide (Costa et al., 2008). In addition, PRX1 has been suggested to act as positive regulator and interacts with ethylene responsive factor (ERF) regulating accumulation of 3′,4′-anhydrovinblastine in *C. roseus* (Wang et al., 2016).

The success of genetic engineering depends on genetic and chemical stability of transgenic plants in subsequent generations (Verma et al., 2015). The copy number of integrated gene affects the expression level and genetic stability in transgeniclines. One or two copies of target gene integration in transgenic plants have been reported ideal for genetic stability (Tang et al., 2007). The integration of multiple copies of transgene into one or more chromosomes might result in low genetic stability, transcriptional or post transcriptional gene silencing. Lately, qPCR has been successfully utilized for determining transgene copy number in transgenic plants of different species (Weng et al., 2004; Yi et al., 2008; Li et al., 2017). In the present study, qPCR determination of copy number revealed that one to two copies of *G(G)PPS* in different *G(G)PPS* and *G(G)PPS+GES* overexpressing lines, whereas it was found to be two for *GES* in all three *G(G)PPS+GES* transgenic lines (Supplementary Table S2). Previous transgenic studies in *C. roseus* plants have analyzed the metabolites in only T_0_ plants (Pan et al., 2012; Wang Q. et al., 2012). Hence to check the chemo-stability in subsequent generations, the level of secologanin and downstream alkaloids were measured in T_1_ lines of *G(G)PPS* and *G(G)PPS*+*GES* transgenic lines. While catharanthine and vindoline displayed better accumulation in two T_1_ *G(G)PPS* lines [*G(G)PPS*_5 and *G(G)PPS*_7] compared to that of T_0_ plants (**Figures 5A**, **7A**), the vinblastine levels were comparable in both T_0_ and T_1_ lines (**Figures 5B**, **7B**). Among the three T_1_ *G(G)PPS*+*GES* lines, *G(G)PPS*+*GES*_1 and *G(G)PPS*+*GES*_2 lines exhibited a similar trend to that of T_0_ plants for all analyzed metabolites including vinblastine (**Figures 5B,F**, **7B,D**). As observed in T_0_ lines, *PRX1* exhibited differential expression that correlated with vinblastine levels in T_1_ transgenic lines with higher levels of vindoline and catharanthine (**Figure 6** and Supplementary Figure S9C).

Overexpression of *GPPS* and some of the terpene synthases redirects metabolic flux toward secondary metabolite biosynthesis, thus lowering the flux for the synthesis of other primary metabolites thereby affecting plant growth. For instance, transgenic tobacco plants expressing snapdragon *GPS.SSU* were dwarf and displayed strong chlorosis with reduced chlorophyll content (Orlova et al., 2009). Also, overexpression of strawberry linalool/nerolidol synthase and taxadiene synthase in *Arabidopsis* resulted in a dwarf phenotype due to reduction in gibberellic acid levels (Aharoni et al., 2003; Besumbes et al., 2004). In contrast, co-expression of peppermint *GPPS.SSU* with different monoterpene synthases enhanced the monoterpene production without affecting plant growth in tobacco (Yin et al., 2017). Here, overexpression of *G(G)PPS* alone or together with *GES* did not alter the *C. roseus* plant phenotype (Supplementary Table S3 and Supplementary Figure S4C). Selected transgenic lines were tested for growth parameters and no differences were found (Supplementary Table S3). As we have overexpressed a bifunctional G(G)PPS, it could have provided higher flux of GGPP and GPP, respectively, required for generalized and specialized metabolism in transgenic lines, thereby not affecting the plant growth. Indeed, two best expressing *G(G)PPS* transgenic lines exhibited a significant increase in the level of total chlorophyll indicating that enhanced GGPP production by the bifunctional enzyme could have resulted in improved chlorophyll content (Supplementary Figure S11). Although overexpression of *Picea abies* bifunctional G(G)PPS resulted in increased accumulation of geranylgeranyl fatty acid esters, chlorophylls and carotenoids remained unaffected (Nagel et al., 2014).

In summary, we demonstrate that the level of monomeric MIAs and pharmaceutically important vinblastine can be enhanced in plants by transgenic overexpression of genes involved in early steps of secoiridoid pathway. While overexpression of bifunctional *G(G)PPS* enhanced both monomeric and dimeric MIAs, co-expression of *G(G)PPS* and *GES* led to increase of monomeric MIAs higher than *G(G)PPS* overexpressors. Improved accumulation of MIAs in transgenic periwinkle without compromising the plant growth indicated the positive role of bifunctional *G(G)PPS* in both generalized and specialized metabolism. Commercial exploitation of *G(G)PPS* transgenic lines could reduce the cost of dimeric alkaloids production and co-expressing lines could be exploited for vindoline and catharanthine production or may be used in molecular breeding approaches to further improve the dimeric alkaloids content. Moreover, future research focusing on stacking of a combination of MIA pathway genes including *PRX1* and transcriptional regulators could enhance the flux resulting in further improvement of alkaloids in *C. roseus*.

## Author Contributions

SK and HS performed the experiments. SK, HS, and DN analyzed the data. DN conceived and coordinated the research. SK and DN wrote the manuscript.

## Conflict of Interest Statement

The authors declare that the research was conducted in the absence of any commercial or financial relationships that could be construed as a potential conflict of interest.

## Footnotes

1 http://www.graphpad.com/quickcalcs/ttest1.cfm
