# Supplementary material for: Terpene Moiety Enhancement by Overexpression of Geranyl(geranyl) Diphosphate Synthase and Geraniol Synthase Elevates Monomeric and Dimeric Monoterpene Indole Alkaloids in Transgenic Catharanthus roseus
Source: Front Plant Sci. 2018 Jul 6;9:942. doi: 10.3389/fpls.2018.00942 (PMC6043680; doi:10.3389/fpls.2018.00942)
Supplement: Supplementary file 1 [file Presentation_1.pdf]

Supplementary material

Terpene Moiety Enhancement by Overexpression of Geranyl(geranyl) Diphosphate Synthase and Geraniol Synthase Elevates Monomeric and Dimeric Monoterpene Indole Alkaloids in Transgenic *Catharanthus roseus*

Sarma Rajeev Kumar, Shilpashree, H.B., and Dinesh A. Nagegowda*
Molecular Plant Biology and Biotechnology Lab,
CSIR-Central Institute of Medicinal and Aromatic Plants, Research Centre,
Bengaluru - 560065, India

*Address correspondence to da.nagegowda@cimap.res.in
Dr. Dinesh A. Nagegowda
Molecular Plant Biology and Biotechnology Lab
CSIR- Central Institute of Medicinal and Aromatic Plants Research Centre,
Allalasandra, GKVK Post, Bengaluru – 560065, India
Telephone: +91-80-28460563
Fax: +91-80-28564707

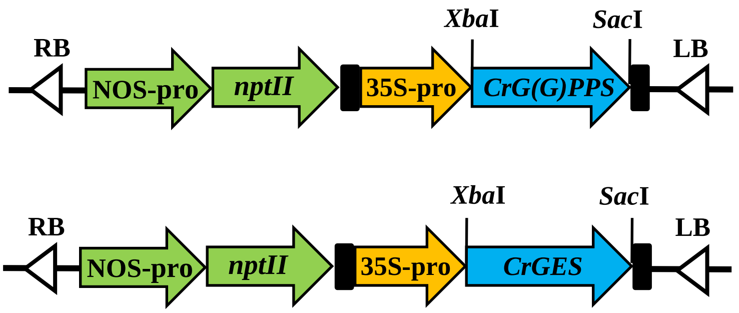

Fig. S1. T- DNA map of pBI121::*G(G)PPS* and pBI121::*GES*

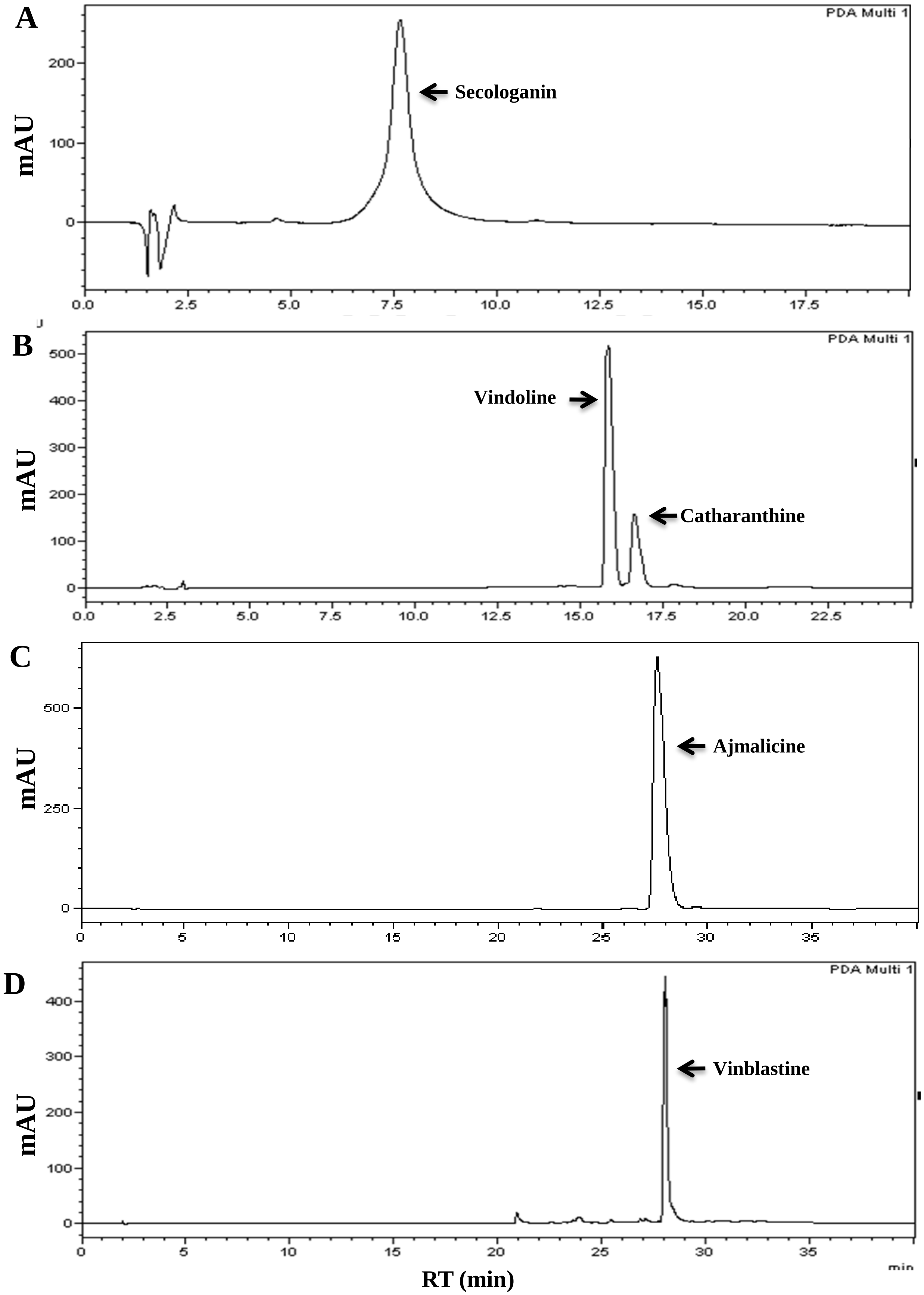

Fig. S2. Standard chromatogram of secologanin (A), vindoline and catharanthine (B), ajmalicine (C) and vinblastine (D). Data were extracted at 238 nm for secologanin, 254 nm for vindoline, catharanthine, ajmalicine and vinblastine.

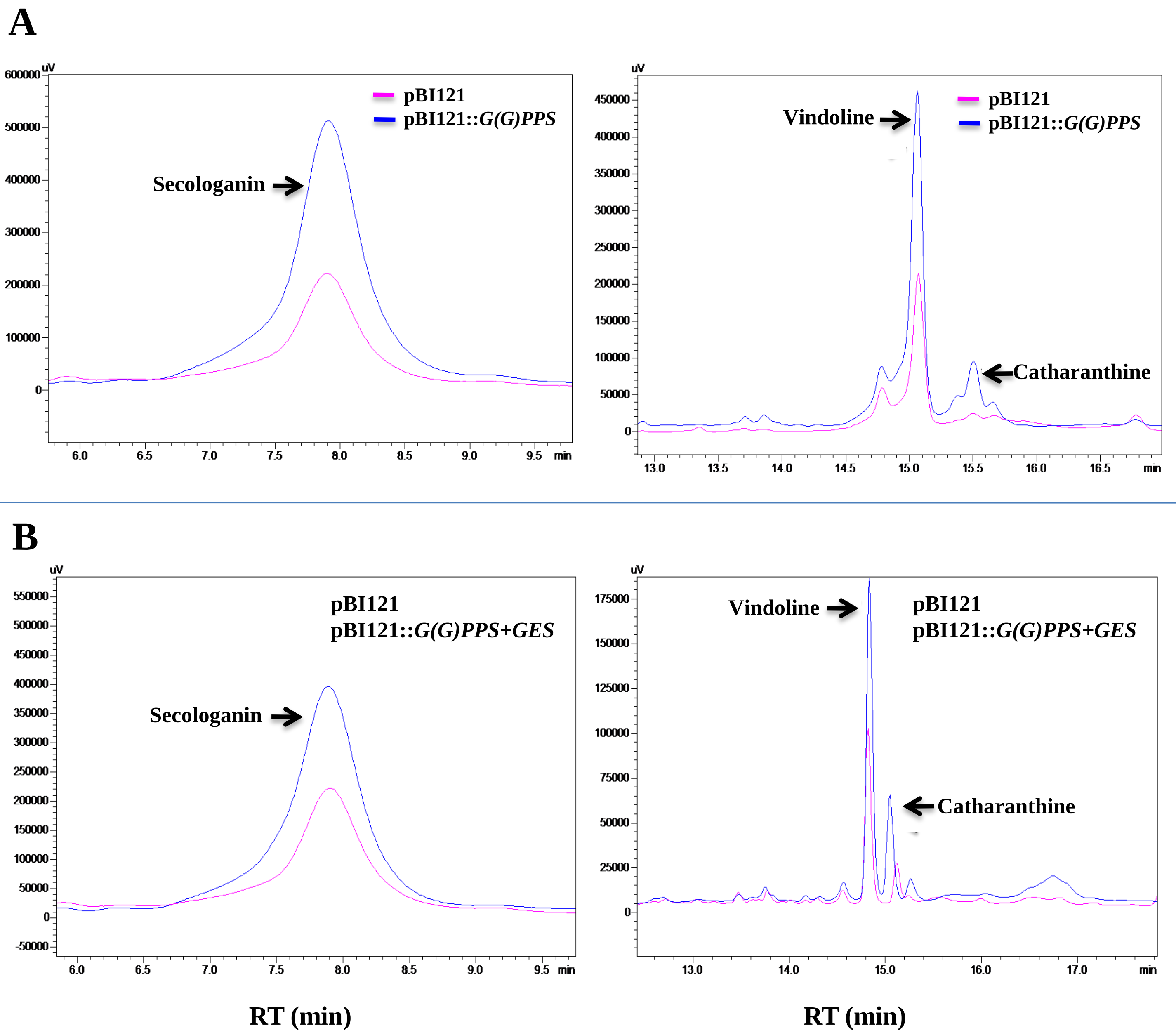

Fig. S3. Representative HPLC chromatograms of secologanin, and vindoline and catharanthine in *C. roseus* leaves transiently overexpressiing *G(G)PPS* (A) and *G(G)PPS+GES* (B)

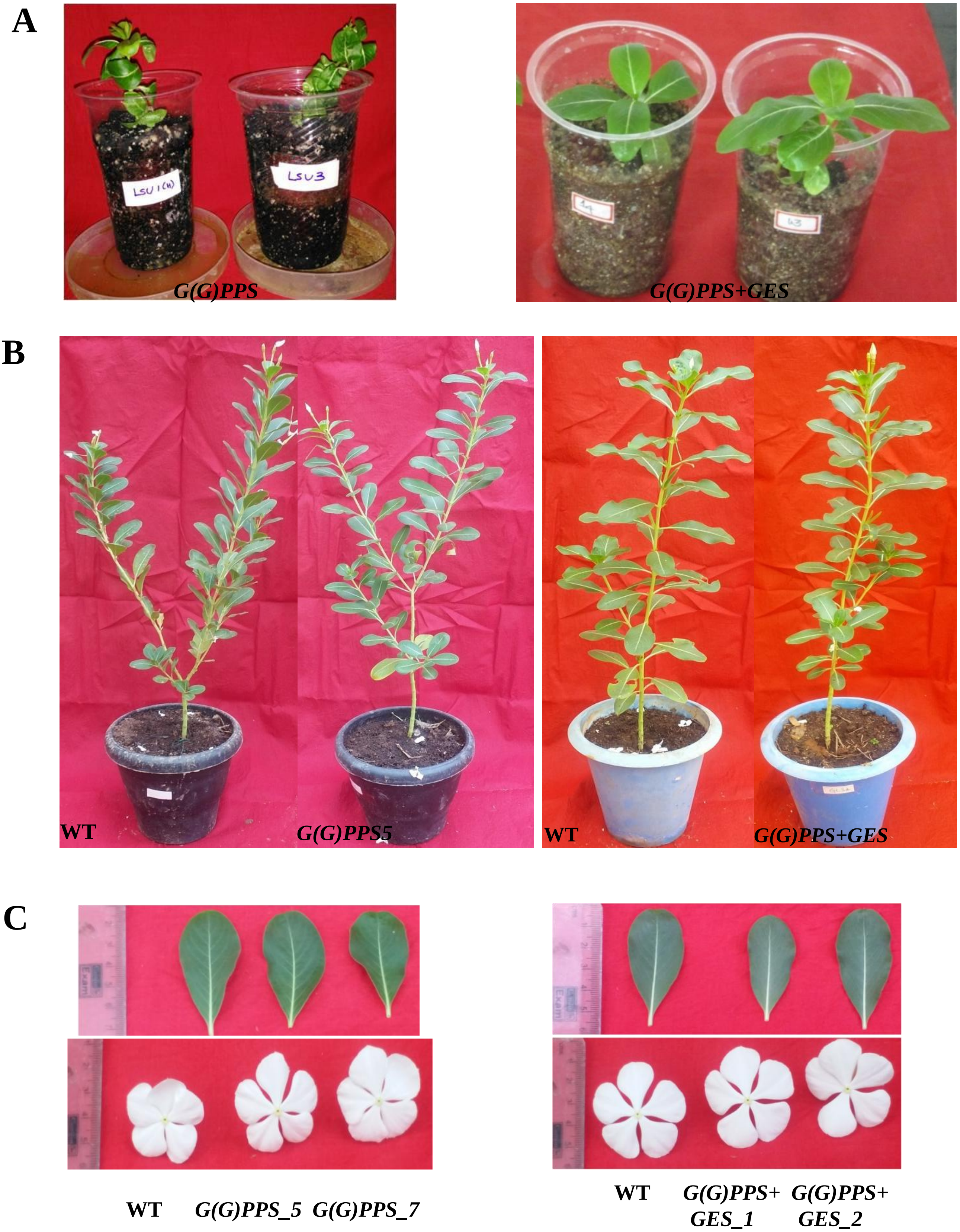

Fig. S4. Hardening and phenotype of transgenic *C. roseus* plants. (A) Representative photos showing hardening of putative transgenic plants. (B) Transgenic plants in glass house. (C) Comparative phenotype of leaves and flowers of *G(G)PPS* and *G(G)PPS+GES* transgenic *C. roseus.*

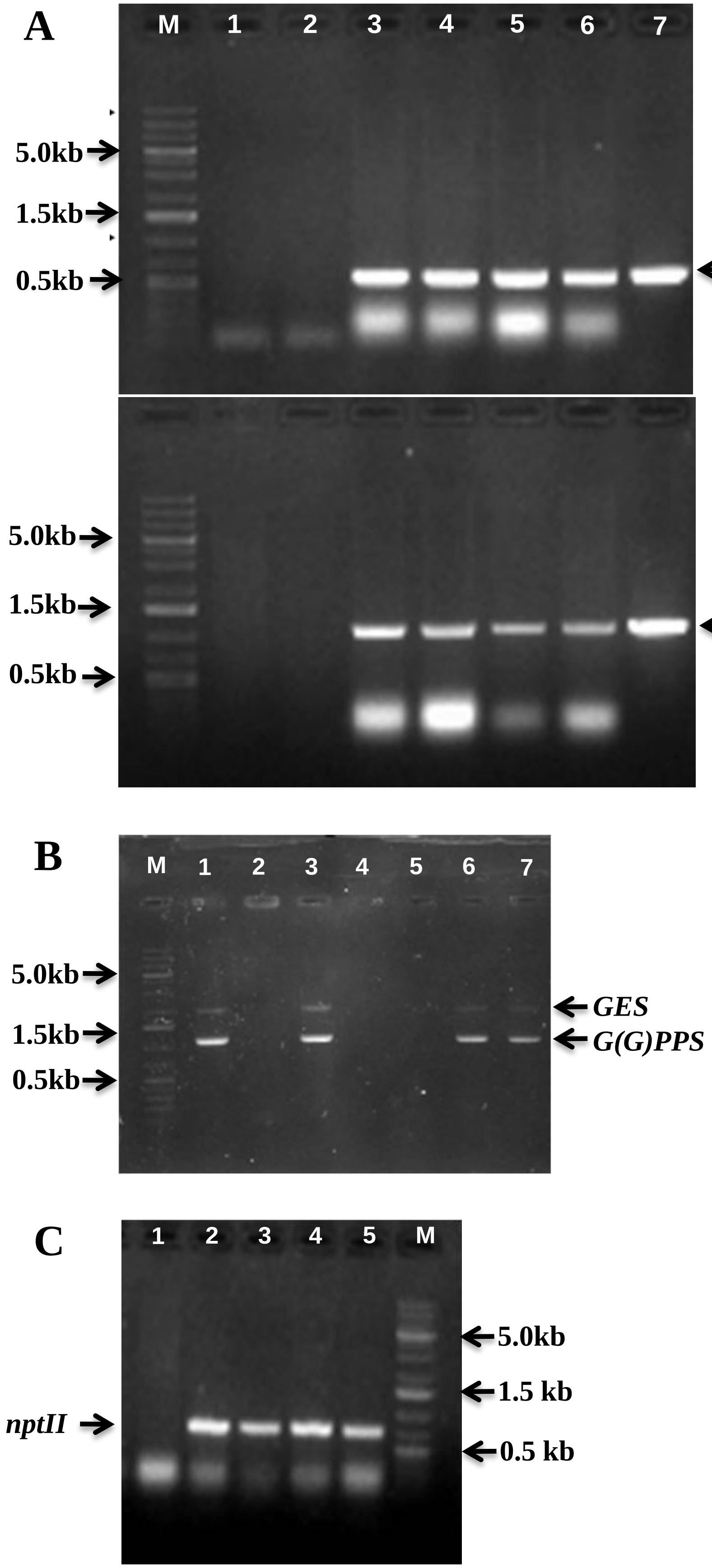

Fig. S5. Screening of putative transgenic *C. roseus* plants. (A) Representative gel image showing PCR screening of T_0 *G(G)PPS* transgenic lines. Upper panel shows PCR using 35S forward and *G(G)PPS* reverse primer 1 and lower panel shows amplification using 35S forward primer and *G(G)PPS* reverse primer 2. Lanes 1 & 2, wild type *C. roseus;* lanes 3 to 6, G(G)PPS positive lines (lines 1 to 4), lane 7, pBI121::G(G)PPS plasmid +ve control. (B) PCR screening of *G(G)PPS+ GES* co-expressing T_0 lines using 35S forward and *G(G)PPS and GES* reverse primers. Lane 1, pBI121::*G(G)PPS+GES* plasmid +ve control; lane 2, wild type *C. roseus*. Lanes 3, 6, & 7 showed amplification of expected sizes confirming the transgenic nature and were named as *G(G)PPS+GES_1*, *G(G)PPS+GES_2* and *G(G)PPS+GES_3* lines. Lanes 4 & 5 did not show any amplification. (C) Reconfirmation of *G(G)PPS+GES* transgenic lines using *nptII* primers. Lane 1, wild type *C. roseus*. Lane 2, pBI121::*G(G)PPS+GES* plasmid +ve control; Lanes 3, 4, & 5 represent *G(G)PPS+GES_1*, *G(G)PPS+GES_2* and *G(G)PPS+GES_3* lines. M, 1 kb plus DNA ladder.

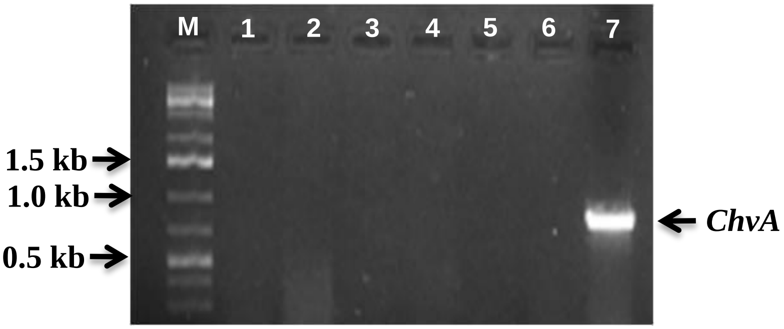

Fig. S6. Representative gel image showing the absence of *Agrobacterium tumefaciens* contamination in transgenic lines. PCR was performed using *A. tumefaciens g*enome specific ChvA (chromosomal virulence gene) forward and reverse primers. Lane 1, wild type C. roseus; lanes 2 to 4, *G(G)PPS* transgenic lines; lanes 5 & 6, *G(G)PPS+GES* transgenic lines; lane 7, Genomic DNA of *A. tumefaciens*.

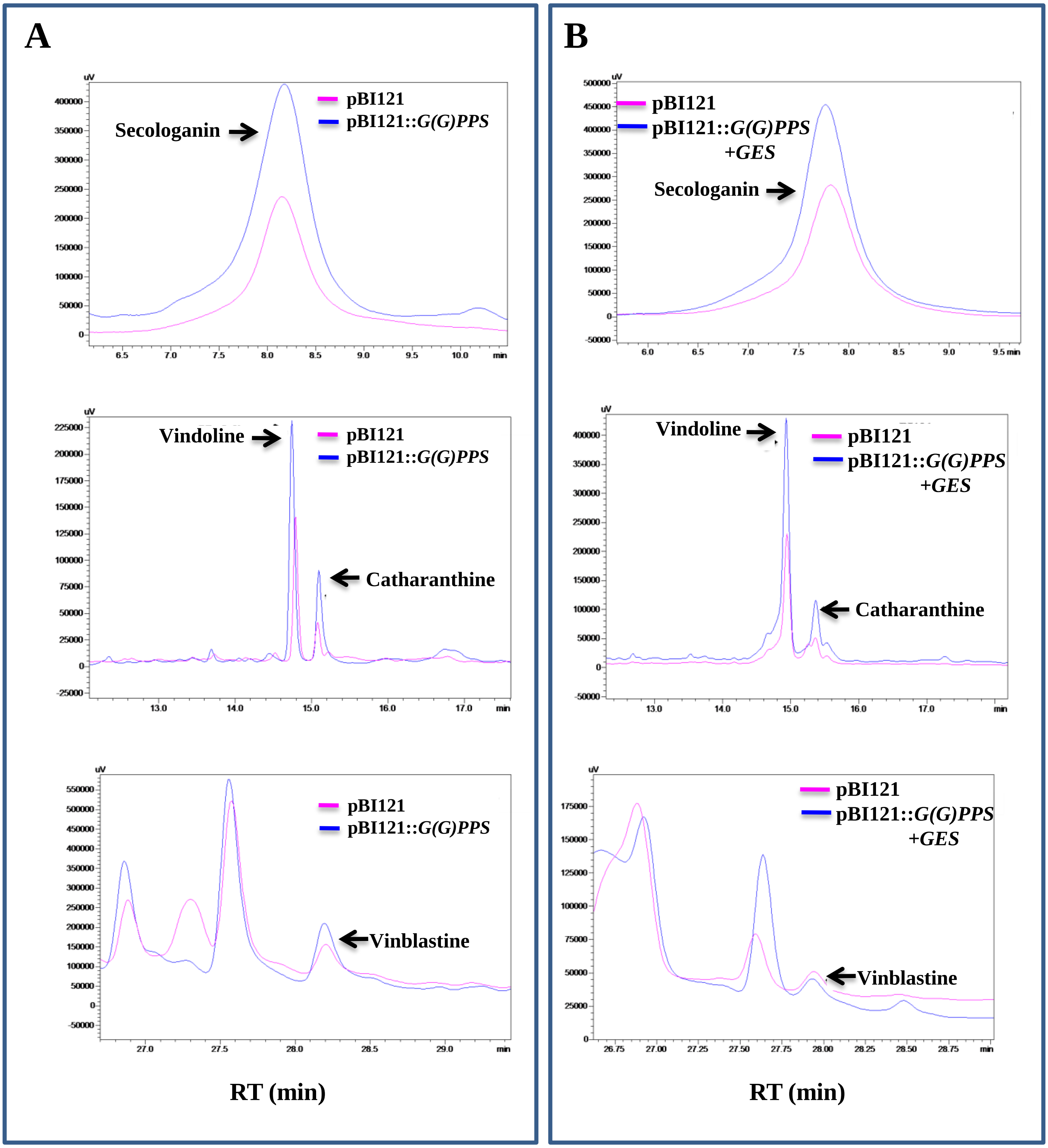

Fig. S7. Representative HPLC chromatograms of secologanin, vindoline, catharanthine and vinblastine in T_0 transgenic lines of *C. roseus* overexpressing *G(G)PPS* (A) and *G(G)PPS+GES* (B).

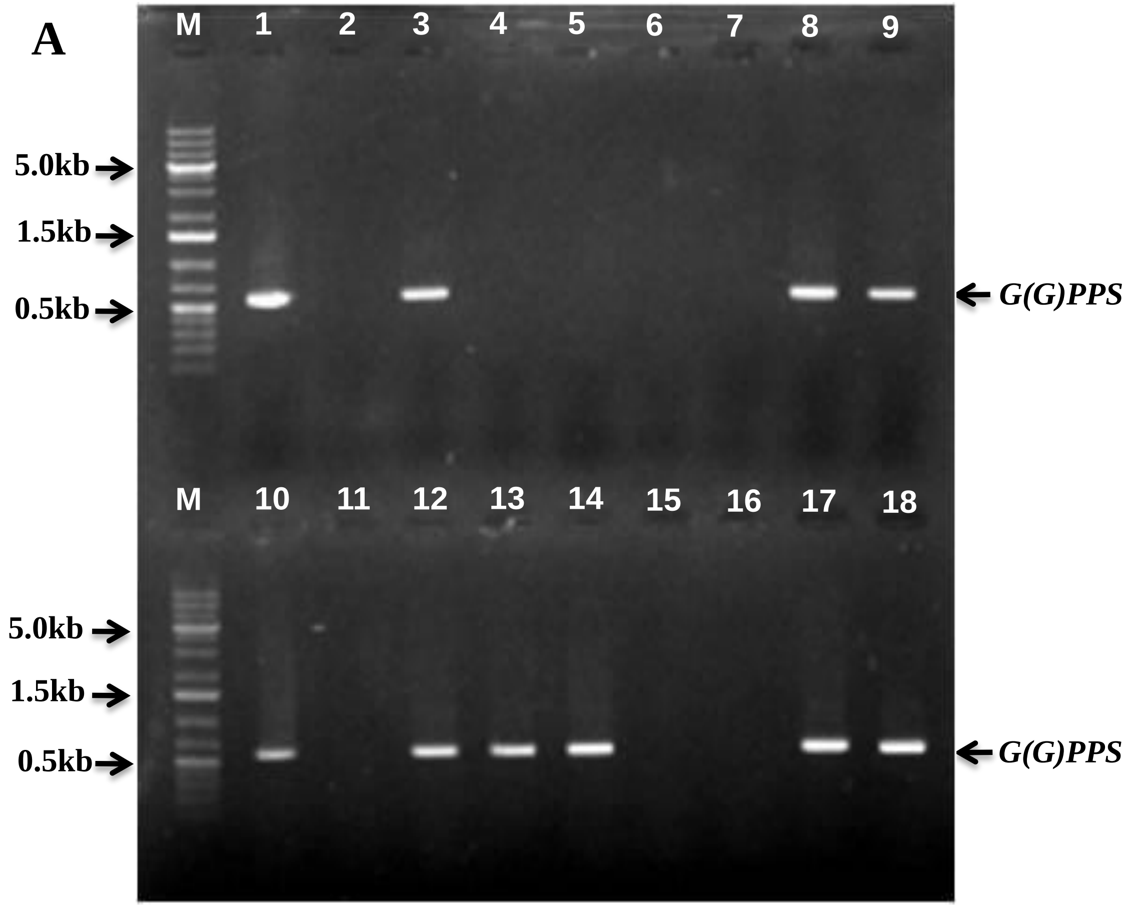

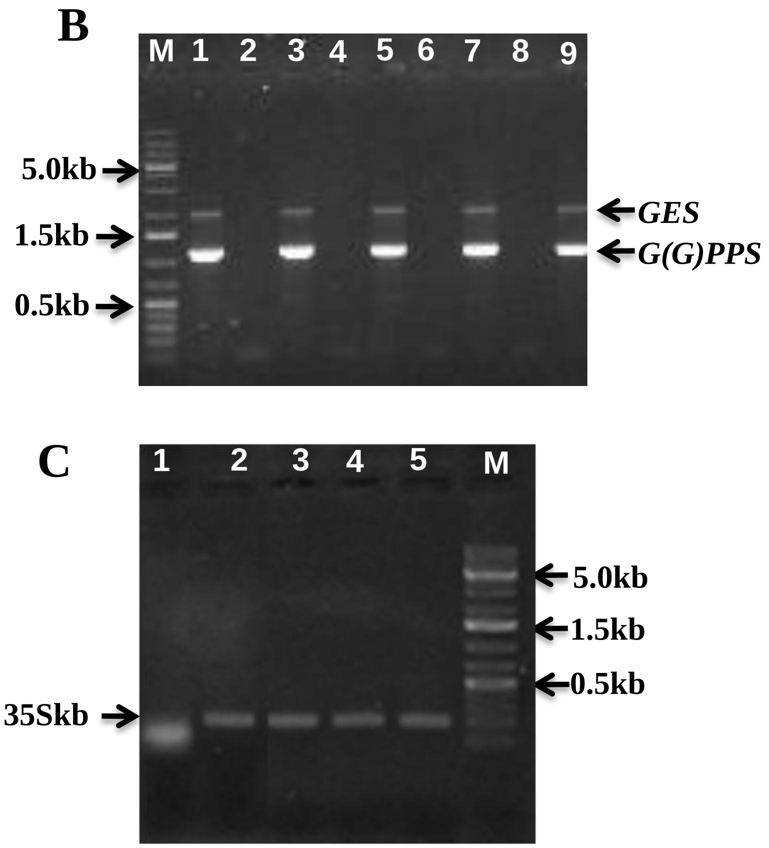

Fig. S8. Screening of transgenic *C. roseus* plants in T_1 generation. (A) Representative gel image showing PCR screening of T1 *G(G)PPS* transgenic lines. PCR amplification using 35S forward and *G(G)PPS* reverse primer 2. Lanes 1 & 2, pBI121::G(G)PPS plasmid +ve control and wild type *C. roseus*, respectively*;* lanes 3, 8-10, 12-14, 17, and 18, respectively corresponds to the amplification in *G(G)PPS_1 to G(G)PPS_7;* lanes 4-7, 11, 15-16 were negative for *G(G)PPS.* (B) PCR screening of *G(G)PPS+GES* co-expressing T_1 lines using *35S* forward and *G(G)PPS* and *GES* reverse primers. Lane 1, pBI121::*G(G)PPS+GES* plasmid +ve control; lane 2, wild type *C. roseus*, lanes 3 & 5, 7, and 9 respectively corresponds to lines *G(G)PPS+GES_1*, *G(G)PPS+GES_2*, and *G(G)PPS+GES_3*. Samples in lanes 4, 6 & 8 did not show any amplification. (C) Reconfirmation of *G(G)PPS+GES* transgenic lines using *CaMV35S* primers. Lane 1, wild type *C. roseus;* lane 2, pBI121::*G(G)PPS+GES* plasmid +ve control; lanes 3, 4, and 5 represent *G(G)PPS+GES_1*, *G(G)PPS+GES_2* and *G(G)PPS+GES_3* lines. M, 1 kb plus DNA ladder.

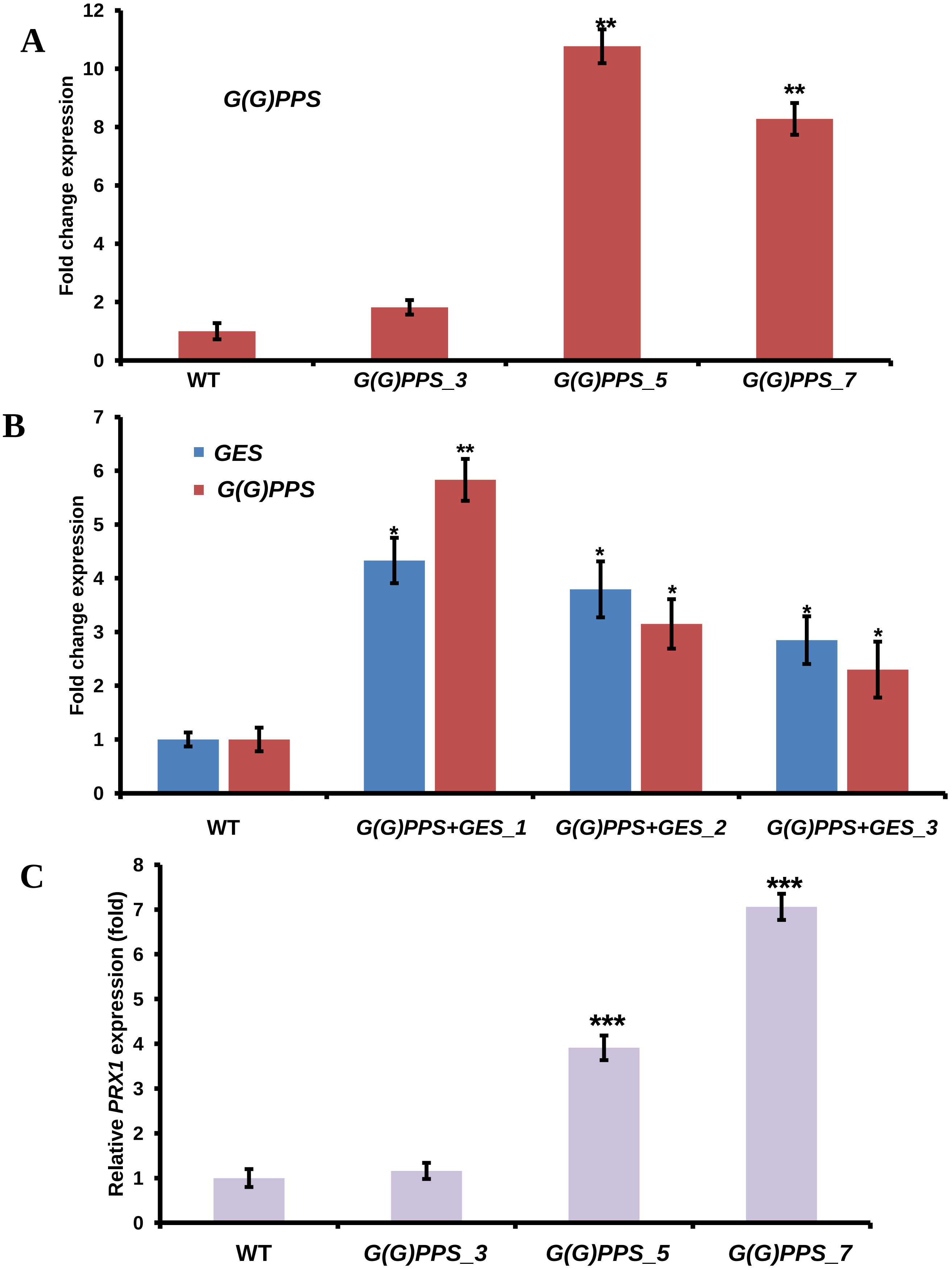

Fig. S9. RT-qPCR analysis of *G(G)PPS* and *GES* in T_1 transgenic lines of *C. roseus* overexpressing *G(G)PPS* (A) and *G(G)PPS+GES* (B). Expression of *PRX1* in T_1 lines of *G(G)PPS* overexpressors (C).

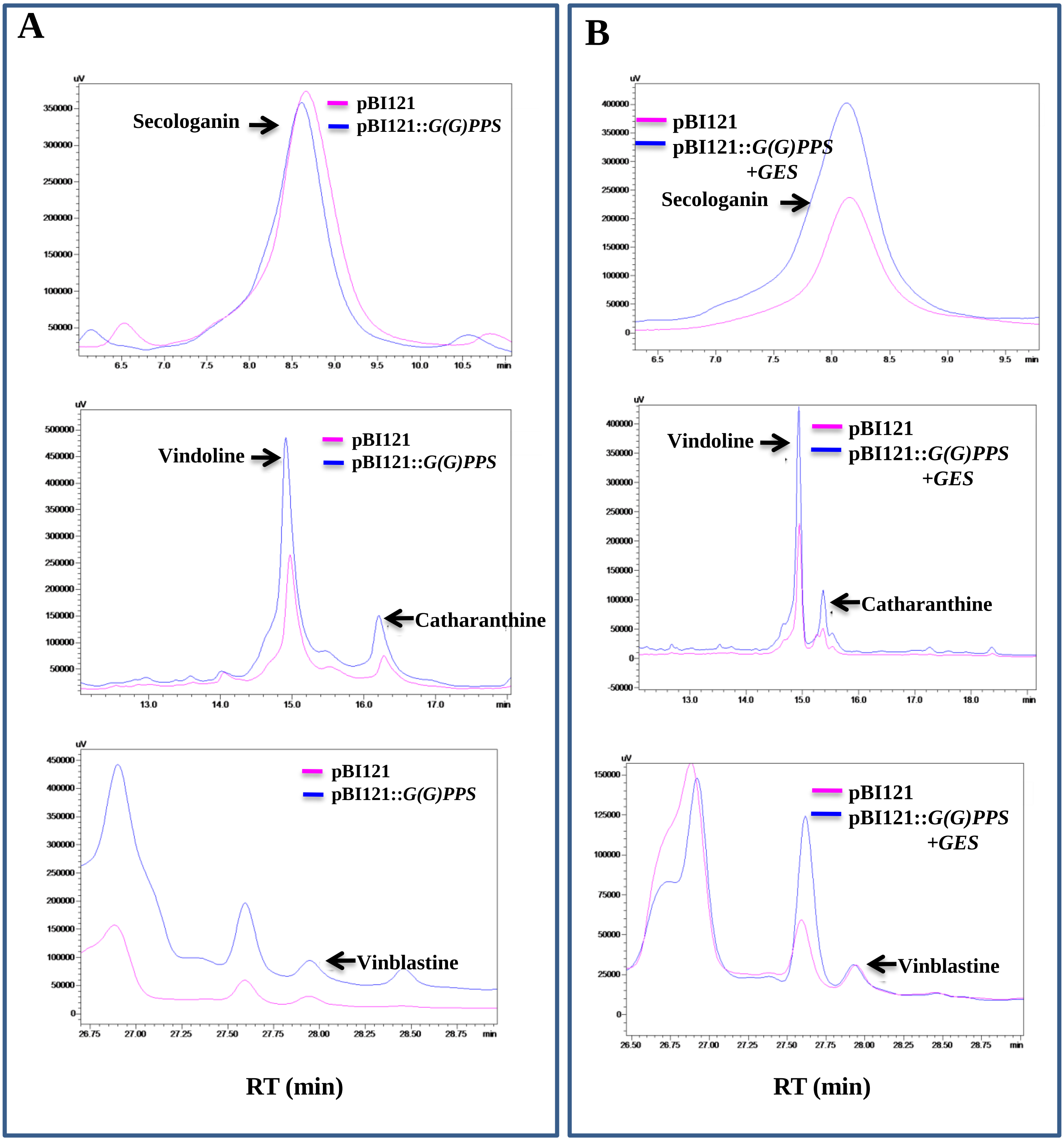

Fig. S10. Representative HPLC chromatogram of secologanin, vindoline, catharanthine and vinblastine in stable transgenic lines $T_1$of *C. roseus* overexpressing *G(G)PPS* (A) and *G(G)PPS+GES* (B)

A

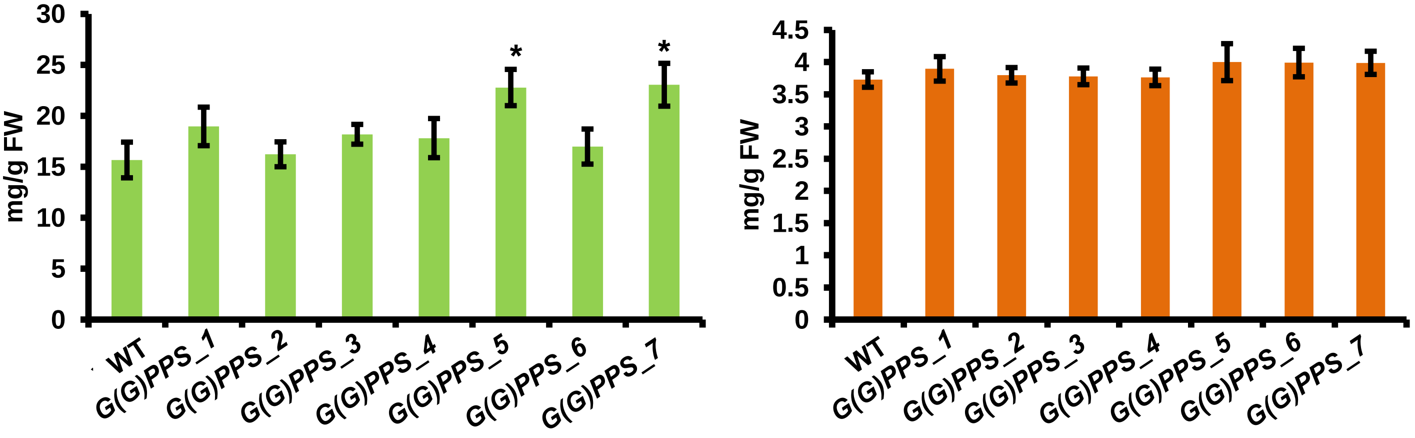

B

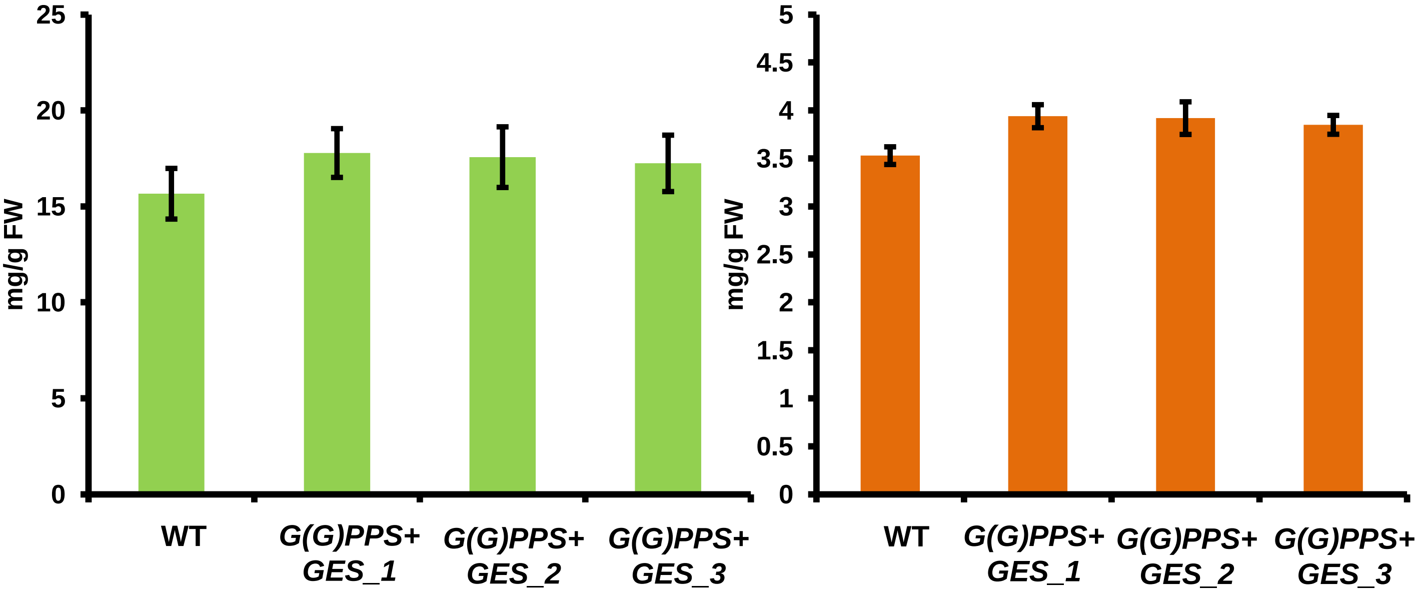

Fig. S11. Analyses of chlorophyll and carotenoids in *C. roseus* overexpressing *G(G)PPS* (**a**) and *G(G)PPS+GES* (B)

Table. S1. Oligonucleotide primer sequences used in the study

**Primer**	**Sequence**	**Purpose**
G(G)PPS Full length F	TCTAGAATGAGCTTTGTGAATTCAATTAC	Full length gene amplification
G(G)PPS Full length R	GAGCTCTTAGTTATCACTGTAGGCTATG	Full length gene amplification
GES Full length R	GAGCTCTTAAAAACAAGGTGTAAAAAACAAAGC	Full length cloning and PCR screening
G(G)PPS Real time F	GATGTGTGCTCTGAGGGCATT	Used for RT-qPCR analysis
G(G)PPS Real time R (internal primer 1)	TTAGCACCACCCACAATTGCC	Used for RT-qPCR analysis and PCR screening
G(G)PPS (internal primer 2)	CGGATCCGGCCTTTCCAATCATATATGCC	Used in transgenic line screening by PCR
GES Real time F	TTGAACCATTCTGCTTCAACCA	Used for RT-qPCR analysis
GES Real time R	TGGTTGGAGTGTTCTTCCTTACC	Used for RT-qPCR analysis
N227 Real time F	TCCTTACGCCGCATTATCAG	Used for RT-qPCR analysis
N227 Real time R	AGATGAGACAGTAACGCCTTG	Used for RT-qPCR analysis
PRX1 Real time F	TGACAGGAGGTCCGGATTATG	Used for RT-qPCR analysis
PRX1 Real time R	TGCTATTGTGTCTGCCCTTGTT	Used for RT-qPCR analysis
CaMV35S F	GCTCCTACAAATGCCATCA	Used for screening transgenic lines by PCR
CaMV35S R	GATAGTGGGATTGTGCGTCA	Used for screening transgenic lines by PCR
ChvA F	CGAAACGCTGTTCGGCCTGTGG	Used to check *Agrobacterium* contamination in transgenic lines
ChvA R	G TTCAGCAGGCCGGCATCCTGG	Used to check *Agrobacterium* contamination in transgenic lines
nptII F	GAAGAACTCGTCAAGAAGGCG	Used for screening transgenic lines by PCR
nptII R	GGAGAGGCTATTCGGCTA	Used for screening transgenic lines by PCR

Table. S2. Analyses of gene copy number in transgenic *C. roseus* lines expressing *G(G)PPS* and *G(G)PPS+GES*

***C. roseus* transgenic line**	**Estimated copy number of *G(G)PPS***	**Actual copy number of transgene**	**Estimated copy number of *GES***	**Actual copy number of transgene**
WT control	1	0	1	0
G(G)PPS_1	2	1	-	-
G(G)PPS_3	2	1	-	-
G(G)PPS_5	3	2	-	-
G(G)PPS_7	2	1	-	-
G(G)PPS+GES_1	3	2	3	2
G(G)PPS+GES_2	2	1	3	2
G(G)PPS+GES_3	2	1	3	2

Table. S3. Analyses of phenotypic parameters in transgenic *G(G)PPS* and *G(G)PPS+GES C. roseus* lines

**15 days after transfer**	No. of Leaf pair	No. of branches	No. of flowers	No. of siliques
**WT1**	4	0	0	0
**WT2**	3	0	0	0
**GPPS_5**	4	0	0	0
**GPPS_7**	4	0	0	0
**GPPS+GES_1**	3	0	0	0
**GPPS+GES_2**	4	0	0	0
**GPPS+GES_3**	3	0	0	0
**30 days after transfer**	No. of Leaf pair	No. of branches	No. of flowers	No. of siliques
**WT1**	8	0	1	0
**WT2**	7	0	0	0
**GPPS_5**	8	0	0	0
**GPPS_7**	8	0	1	0
**GPPS+GES_1**	7	0	0	0
**GPPS+GES_2**	7	0	1	0
**GPPS+GES_3**	7	0	0	0
**45 days after transfer**	No. of Leaf pair	No. of branches	No. of flowers	No. of siliques
**WT1**	11	1	10	1
**WT2**	12	0	11	0
**GPPS_5**	11	1	12	1
**GPPS_7**	11	1	11	1
**GPPS+GES_1**	12	1	13	0
**GPPS+GES_2**	12	1	12	1
**GPPS+GES_3**	11	0	11	0
**60 days after transfer**	No. of Leaf pair	No. of branches	No. of flowers	No. of siliques
**WT1**	13	2	21	10
**WT2**	16	2	22	8
**GPPS_5**	14	2	19	11
**GPPS_7**	13	3	22	12
**GPPS+GES_1**	14	3	25	13
**GPPS+GES_2**	16	2	24	11
**GPPS+GES_3**	16	3	21	9
